# Blockade of Stromal Gas6 Alters Cancer Cell Plasticity, Activates NK Cells, and Inhibits Pancreatic Cancer Metastasis

**DOI:** 10.3389/fimmu.2020.00297

**Published:** 2020-02-27

**Authors:** Lucy Ireland, Teifion Luckett, Michael C. Schmid, Ainhoa Mielgo

**Affiliations:** Department of Molecular and Clinical Cancer Medicine, University of Liverpool, Liverpool, United Kingdom

**Keywords:** Gas6, pancreatic cancer, metastasis, macrophages, fibroblasts, NK cells

## Abstract

Pancreatic ductal adenocarcinoma (PDA) is one of the deadliest cancers due to its aggressive and metastatic nature. PDA is characterized by a rich tumor stroma with abundant macrophages, fibroblasts, and collagen deposition that can represent up to 90% of the tumor mass. Activation of the tyrosine kinase receptor AXL and expression of its ligand growth arrest-specific protein 6 (Gas6) correlate with a poor prognosis and increased metastasis in pancreatic cancer patients. Gas6 is a multifunctional protein that can be secreted by several cell types and regulates multiple processes, including cancer cell plasticity, angiogenesis, and immune cell functions. However, the role of Gas6 in pancreatic cancer metastasis has not been fully investigated. In these studies we find that, in pancreatic tumors, Gas6 is mainly produced by tumor associated macrophages (TAMs) and cancer associated fibroblasts (CAFs) and that pharmacological blockade of Gas6 signaling partially reverses epithelial-to-mesenchymal transition (EMT) of tumor cells and supports NK cell activation, thereby inhibiting pancreatic cancer metastasis. Our data suggest that Gas6 simultaneously acts on both the tumor cells and the NK cells to support pancreatic cancer metastasis. This study supports the rationale for targeting Gas6 in pancreatic cancer and use of NK cells as a potential biomarker for response to anti-Gas6 therapy.

## Introduction

Growth arrest-specific gene 6 (Gas6) is a multifunctional factor that regulates several processes in normal physiology and pathophysiology (1). Gas6 binds to the Tyro3, Axl, and Mer (TAM) family of receptor tyrosine kinases (TAM receptors) with the highest affinity for Axl (2). Gas6 supports erythropoiesis, platelet aggregation, angiogenesis, efferocytosis and inhibits the immune response (3). Gas6 is critical for the maintenance of immune homeostasis and mice deficient in Gas6 or TAM receptors experience severe autoimmune diseases (4). Gas6 and its main receptor Axl are overexpressed in several cancer types including, breast, ovarian, gastric, glioblastoma, lung, and pancreatic cancer and their expression correlates with a poor prognosis (5). Axl is ubiquitously expressed in all tissues (6) but is particularly notable in cancer cells, macrophages, dendritic cells and natural killer cells for its role in driving immunosuppression and tumor progression (7–9). Several cancer studies have focused on the role of Gas6-Axl signaling on the tumor cells and have demonstrated that Axl activation supports tumor cell proliferation, epithelial-mesenchymal transition (EMT), drug resistance, migration and metastasis (5). Factors secreted within the tumor microenvironment are able to sustain Gas6/Axl signaling. Hypoxia Inducible Factor (HIF) has been shown to bind to the *Axl* promoter region and upregulate its expression on renal cell carcinoma cells (10). Secretion of IL-10 and M-CSF by tumor cells induces tumor associated macrophages to secrete Gas6 (11). However, only a few studies have investigated the role of Gas6-Axl signaling in the immune response to breast cancer, ovarian cancer and melanoma (7, 9).

In solid tumors such as breast or pancreatic cancer, the tumor stroma can represent up to 80% of the tumor mass and actively influences cancer progression, metastasis (12–14) and resistance to therapies (15–17).

Pancreatic ductal adenocarcinoma (PDA) is one of the most lethal cancers worldwide and better therapies are urgently needed (18). Metastasis, therapy resistance, and immunosuppression are key characteristics of pancreatic tumors (19, 20). The Gas6–Axl pathway is activated in 70% of pancreatic cancer patients (21) and is associated with a poor prognosis and increased frequency of distant metastasis (22). Blocking Gas6-Axl signaling inhibits cancer progression (23, 24) and several Axl inhibitors and warfarin (a vitamin K antagonist that blocks Gas6 signaling) are currently being tested in cancer patients, including PDA patients. While the cancer cell autonomous functions of Gas6 are well-documented, the effect of Gas6 signaling in the stroma/immune compartment in pancreatic cancer has not been fully explored. In these studies, we sought to understand the effect of Gas6 blockade in both the tumor and the stroma/immune compartments, *in vivo*, in pancreatic cancer. Gaining a better understanding of how blockade of Gas6 signaling affects pancreatic cancer is important because it will help design and interpret the results of the recently launched clinical trials that are testing anti-Gas6/TAM receptors therapies in pancreatic cancer patients (25).

## Results

### Pharmacological Blockade of Gas6 Inhibits Spontaneous Pancreatic Cancer Metastasis

**Figure 1:**
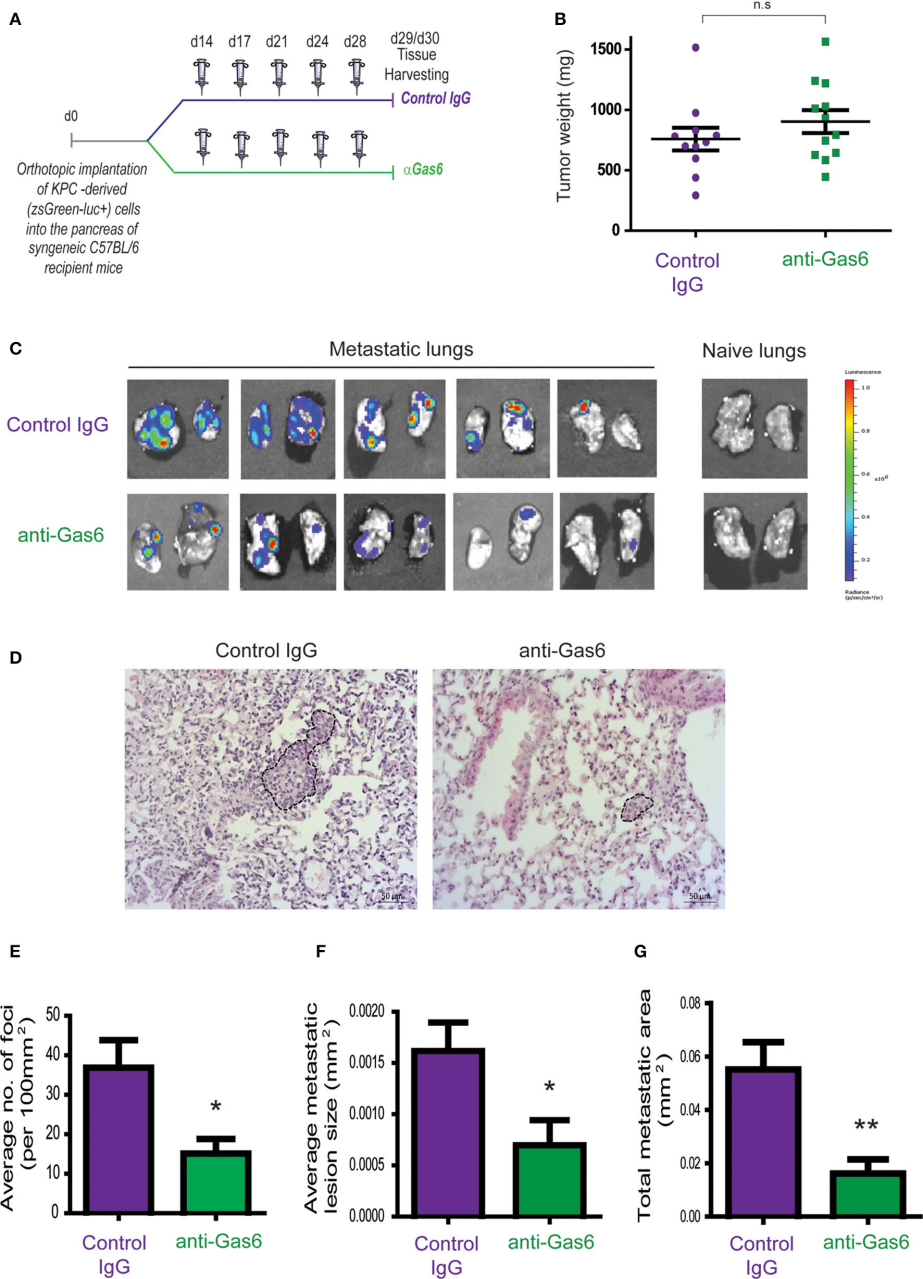
Pharmacological blockade of Gas6 inhibits pancreatic cancer metastasis. **(A)** KPC^luc/zsGreen^ (zsGreen) -derived pancreatic tumor cells (FC1242^luc/zsGreen^) were orthotopically implanted into the pancreas of syngeneic C57BL/6 recipient mice, and mice were treated, starting at day 14 after tumor implantation, twice a week i.p., with either isotype control IgG antibody or Gas6 blocking antibody (2 mg/kg). Primary pancreatic tumors, livers, lungs, and mesenteric lymph nodes were harvested at day 30. **(B)** Tumor weights (*n* = 11 mice for control IgG treatment group; *n* = 12 mice for anti-Gas6 treatment groups). **(C)** Representative IVIS images of metastatic lungs from control IgG and anti-Gas6 treated mice. **(D)** Representative images of H&E staining of metastatic lungs from control IgG and anti-Gas6 treated mice. Scale bar 50 μm. **(E)** Quantification of number of lung metastatic foci per 100 mm^2^ in mice treated with control IgG or anti-Gas6 antibody identified by H&E. *p ≤ 0.05, using unpaired student *T*-test, error bars represent SEM (*n* = 7). **(F)** Average size of pulmonary metastatic lesions in mice treated with control IgG or anti-Gas6 antibody identified by H&E. *p ≤ 0.05, using unpaired student *T*-test, error bars represent SEM (*n* = 7). **(G)** Quantification of total metastatic burden in mice treated with control IgG or anti-Gas6 antibody identified by H&E. **p ≤ 0.01, using unpaired student *T*-test, error bars represent SEM (*n* = 7).

To investigate the effect of Gas6 blockade in pancreatic cancer growth and metastasis, we used an orthotopic syngeneic pancreatic cancer model, in which pancreatic cancer cells derived from the gold standard genetic mouse model of pancreatic cancer (LSL-Kras^G12D^; LSL-Trp53^R172H^; Pdx1-Cre mice; KPC model), transduced with a reporter lentivirus expressing zsGreen/luciferase, were orthotopically implanted into the pancreas of syngeneic immuno-competent mice. This model faithfully recapitulates features of the human disease, and tumors are highly infiltrated by macrophages and are rich in fibroblasts (16, 26, 27). Importantly, pancreatic tumors from this mouse model also showed expression and activation of Axl receptor (Supplementary Figure 1A). These mice were then treated with isotype control IgG antibody or an anti-Gas6 neutralizing antibody (Figure 1A). This anti-Gas6 neutralizing antibody has previously been shown to block Gas6 signaling through the AXL receptor to a similar extent as an anti-AXL antibody (28). Thirty days after implantation, pancreatic tumors, lungs, livers, and mesenteric lymph nodes were surgically removed and analyzed. As expected, control treated mice showed high levels of Axl receptor activation in tumors, whereas the anti-Gas6 treated group showed markedly reduced levels of Axl receptor activation, confirming that anti-Gas6 antibody has reached the tumor and has blocked Axl signaling (Supplementary Figures 1A,B). No differences were seen in primary pancreatic tumor growth (Figure 1B) between the control and anti-Gas6 treatment groups. However, mice treated with the anti-Gas6 antibody showed reduced metastasis to lungs, livers, and mesenteric lymph nodes, compared to control treated mice, as assessed by biolumiscence *ex-vivo* imaging of these organs (Supplementary Figures 1C,D). Since lungs showed the highest level of metastasis in this model, lung tissues were further assessed for metastasis by H&E and cytokeratin 19 (CK19) staining. We observed that both the number of metastatic foci, as well as the size of the metastatic lesions were significantly reduced in control vs. anti-Gas6 treated mice (Figures 1D,E, Supplementary Figures 1E–G). As a consequence the overall metastatic burden was very significantly reduced in the mice treated with anti-Gas6 blocking antibody compared to control mice (Figure 1F). These data suggest that blockade of Gas6 affects the metastatic cascade at different stages, affecting the metastatic spreading and/or initial seeding as well as the metastatic outgrowth of disseminated pancreatic cancer cells.

### Tumor Associated Macrophages and Fibroblasts Are the Main Sources of Gas6 in Pancreatic Cancer

**Figure 2:**
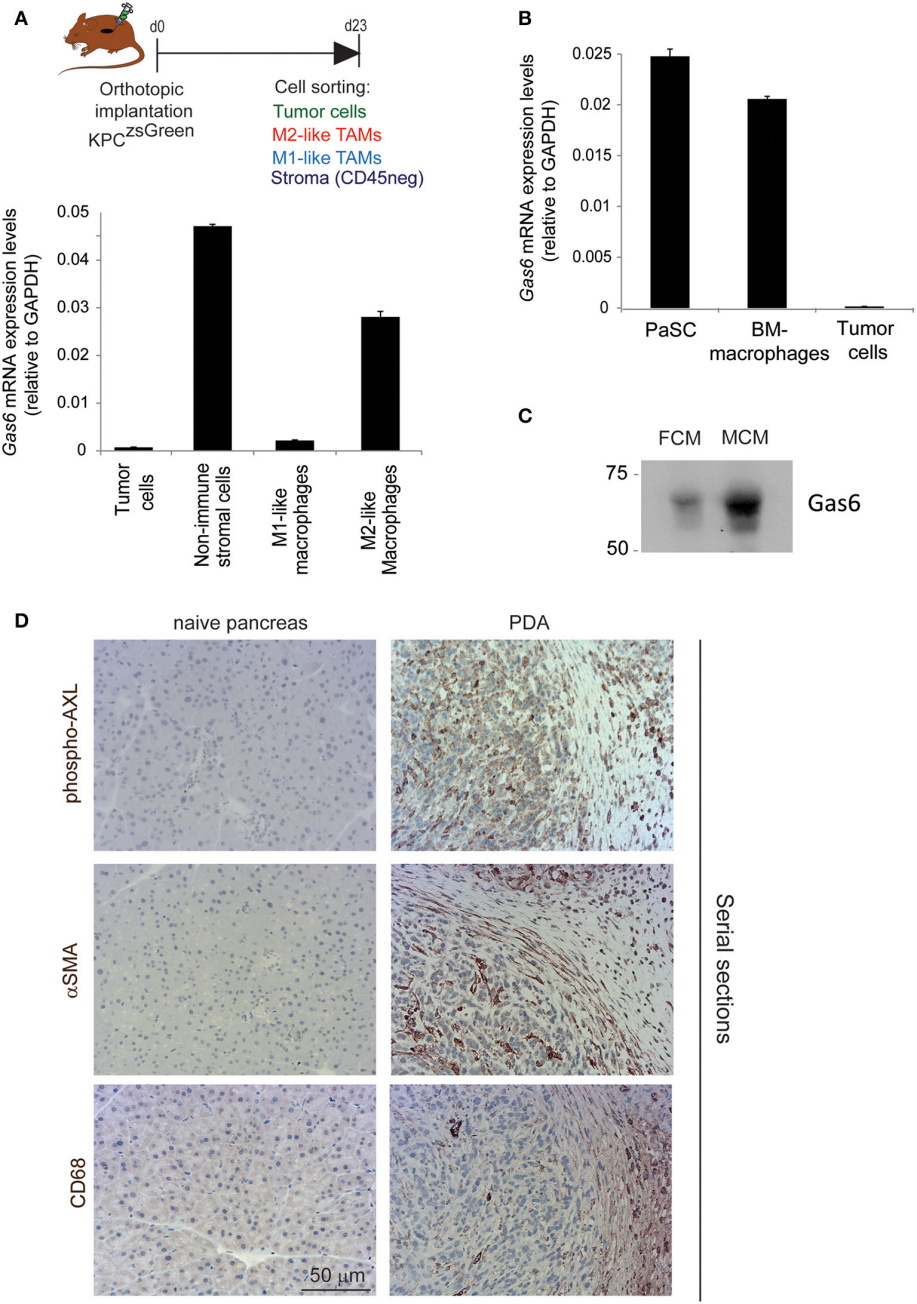
TAMs and CAFs are the main sources of Gas6 in pancreatic tumors. **(A)** KPC^luc/zsGreen^ (zsGreen) -derived tumor cells (FC1242^luc/zsGreen^) were orthotopically implanted into the pancreas of syngeneic recipient (C57/BL6) mice. Tumors were harvested and digested at day 23 after implantation and tumor cells, non-immune stromal cells, M1-like and M2-like macrophages were sorted by flow cytometry. *Gas6* mRNA levels were quantified in CD45–/zsGreen+ tumor cells, CD45–/zsGreen– non-immune stromal cells, CD45+/F4/80+/CD206– M1-like macrophages and CD45+/F4/80+/CD206+ M2-like macrophages sorted by flow cytometry from murine pancreatic tumors. Values shown are the mean and SD (*n* = 3). **(B)** Quantification of *Gas6* mRNA expression levels in *ex vivo* mouse primary isolated macrophages and pancreatic fibroblasts from naïve mice. Values shown are the mean and SD (*n* = 3). **(C)** Immunoblotting analysis of Gas6 secreted protein present in mouse macrophage conditioned media (MCM) and pancreatic fibroblast conditioned media (FCM). **(D)** Images show phospho-Axl, αSMA (fibroblast marker) and CD68 (pan-macrophage marker) staining in naïve mouse pancreas and in serial sections of mouse PDA tissues. Scale bar = 50 μm.

**Figure 3:**
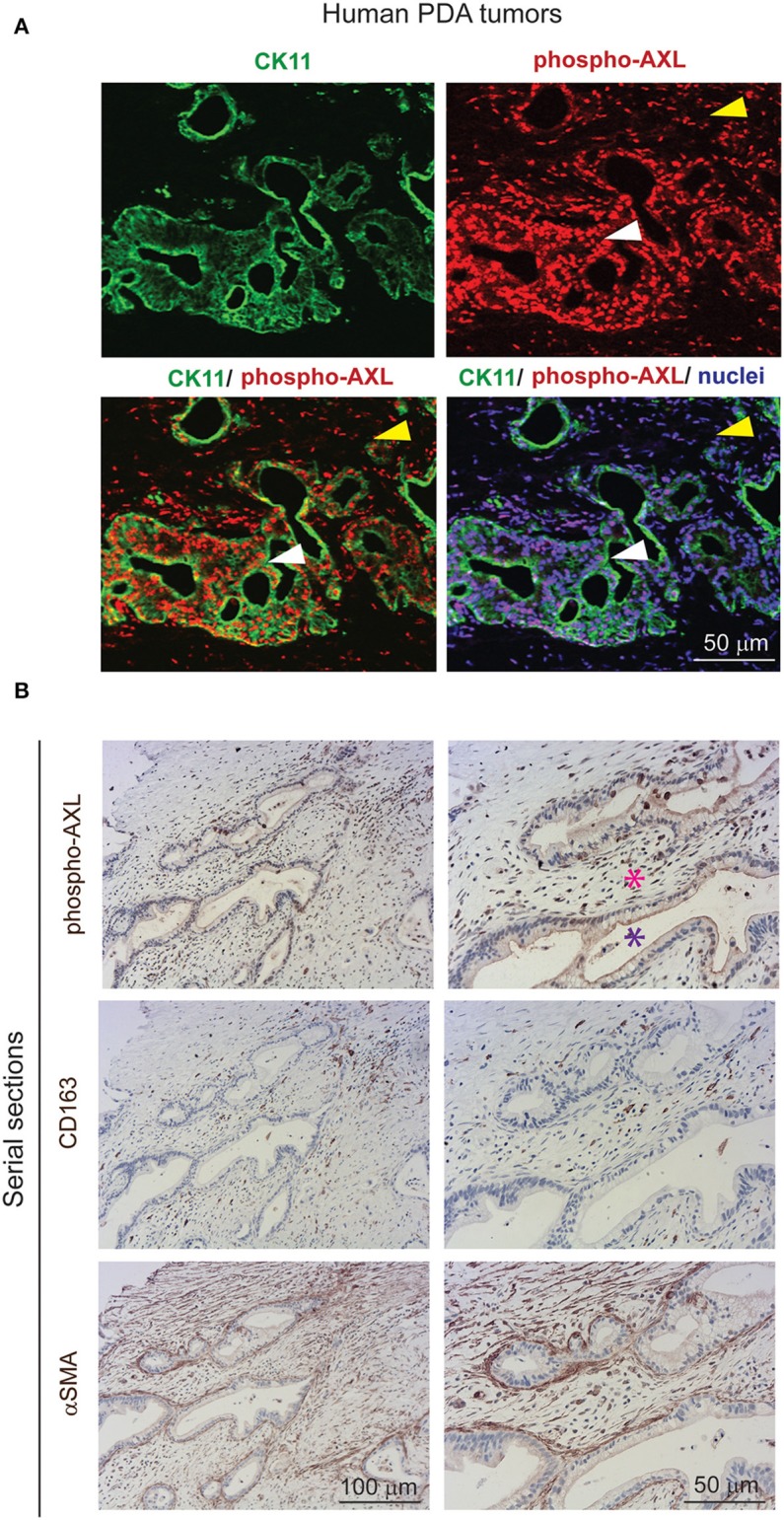
AXL receptor is activated in both the tumor and stromal compartment in biopsies from PDA patients. **(A)** Immunofluorescent staining of human PDA biopsies with CK11 (tumor cell marker, in green), phospho-Axl receptor (in red), and nuclei (in blue). Scale bar, 50 μm. Yellow arrow indicates presence of phosphorylated Axl in the stromal compartment. White arrow indicates presence of phosphorylated Axl in the tumor cells. **(B)** Serial sections of biopsies from human PDA samples immunohistochemically stained for phospho-Axl, CD163 (macrophages) and αSMA (fibroblasts). Cancer cells are indicated by a purple asterisk and tumor stroma is indicated by a pink asterisk. Scale bars, 50 and 100 μm.

Gas6 is a multifunctional protein that is secreted by different cell types. Gas6 has been shown to be produced by macrophages in pre-malignant lesions of a mammary tumor model (29) and in xenograft and orthotopic models of colon and pancreatic cancer (11). Gas6 can also be produced by tumor cells (30) and fibroblasts (31). To determine which cell types produce Gas6 in pancreatic tumors, tumors were harvested at day 23, and tumor cells (CD45–/zsGreen+), non-immune stromal cells (CD45–/zsGreen–), M1-like macrophages (CD45+/F4/80+/CD206–) and M2-like macrophages (CD45+/F4/80+/CD206+) were isolated by flow cytometry (Figure 2A, Supplementary Figure 2A) and analyzed for the expression of *gas6* (Figures 2A,B). We found that both F4/80+/CD206+ (M2-like macrophages) and αSMA+ stromal cells (Supplementary Figure 2B) are the main sources of Gas6 in pancreatic tumors (Figure 2B). *Ex-vivo*, bone-marrow derived macrophages and pancreatic fibroblasts also produce Gas6 (Figures 2B,C). In agreement with these findings, we observed that tumor areas with activated Axl receptor were often surrounded by TAMs and CAFs (Figure 2D). Analysis of Axl expression and activation in pancreatic cancer patient samples has been correlated with a poor prognosis (21, 22) and Axl activation in cancer cells has been shown to support EMT, cell proliferation, metastasis and drug resistance (5). While these studies have mainly focused on analyzing the expression and function of Axl on the cancer cells, Axl is also expressed in immune cells, endothelial cells and stromal cells and regulates innate immunity (3, 4), angiogenesis (32–34), and fibrosis (31). In agreement with this multi-functional role for Axl, we found that Axl is activated in both the tumor and the stromal/immune compartment in biopsies from pancreatic cancer patients (Figures 3A,B).

### Gas6 Blockade Alters EMT of Pancreatic Cancer Cells but Does Not Affect Angiogenesis or Collagen Deposition in Pancreatic Tumors

**Figure 4:**
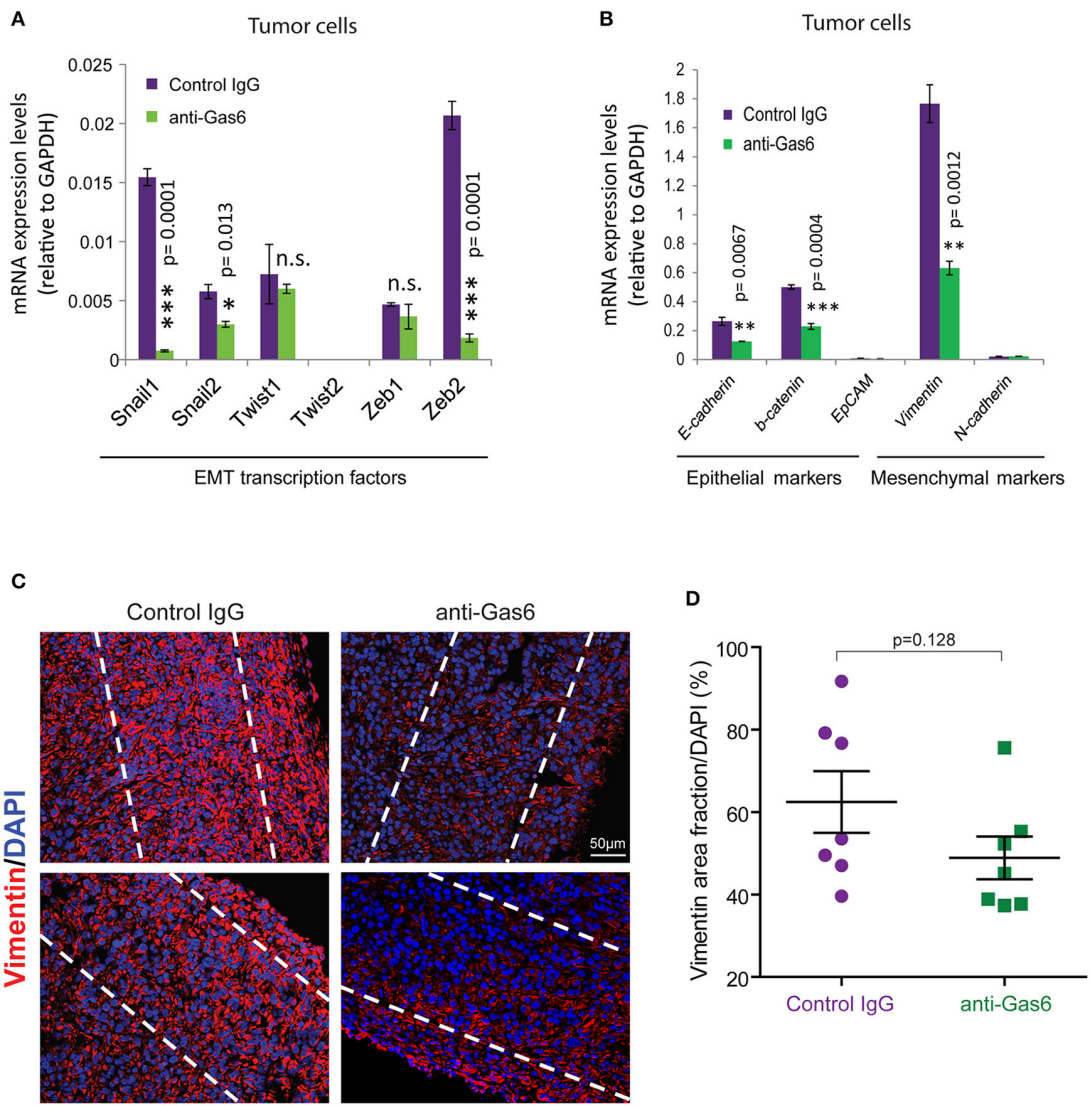
Gas6 blockade in pancreatic tumors partially affects EMT of tumor cells. **(A)** Quantification of the expression levels of the EMT transcription factors: *Snail 1, Snail 2, Twist 1, Twist 2, Zeb 1, and Zeb 2* in tumor cells (zsGreen+) isolated by flow cytometry from mouse PDA tumors. Values shown are the mean and SD (*n* = 3). **(B)** Quantification of the expression levels of the epithelial markers: *E-cadherin, b-catenin, EpCAM*, and the mesenchymal markers *vimentin and N-cadherin* in tumor cells FACS sorted from mouse PDA tumors. Values shown are the mean and SD (*n* = 3). *p ≤ 0.05, using unpaired student *T*-test; **p ≤ 0.01, using unpaired student *T*-test; ***p ≤ 0.005, using unpaired student *T*-test. **(C)** Representative immunofluorescent images of vimentin staining at the periphery of mouse pancreatic tumors treated with control IgG or anti-Gas6 antibody. The dashed lines highlight the areas quantified in the tumor tissues. **(D)** Quantification of vimentin protein expression levels in pancreatic cancer cells. Data are displayed as mean and SEM and represent 5 images per mouse, with 7 animals per treatment group. n.s. no statistically significant differences, using unpaired student *T*-test.

Previous studies have shown that Gas6-Axl signaling promotes tumor cells' EMT (35, 36). To determine whether the reduced metastasis observed when we block Gas6 was caused by an effect on tumor cell EMT, we evaluated the expression of EMT markers and transcription factors on tumor cells from pancreatic tumors treated with isotype control antibody or Gas6 blocking antibody. Tumor cells isolated from pancreatic tumors were analyzed for the expression of the EMT transcription factors *Snail 1, Snail 2, Twist 1, Twist 2, Zeb 1, and Zeb 2* (Figure 4A), the epithelial markers *E-cadherin, b-catenin*, and *Epcam* and the mesenchymal markers *Vimentin* and *N-cadherin* (Figure 4B). We found that blocking of Gas6 significantly decreased the expression of the EMT transcription factors *Snail 1, Snail 2*, and *Zeb 2*, while *twist 1* and *Zeb 1* levels remained unchanged and *twist 2* was not expressed in pancreatic cancer cells (Figure 4A). In agreement with this observation, Gas6 blockade also decreased the expression of the mesenchymal marker *Vimentin*, while *N-cadherin* levels were very low and remained unchanged*. E-cadherin* and *B-catenin* levels were also decreased though upon anti-Gas6 treatment, suggesting that Gas6 signaling partially regulates cancer cell plasticity, a phenomenon previously described in cancer (37, 38). Kirane et al. (23) previously showed that blocking Gas6 signaling with warfarin decreases vimentin expression in a xenograft model of pancreatic cancer.

To further investigate the effect of anti-Gas6 on vimentin expression in pancreatic cancer cells in our *in vivo* tumor model, we analyzed vimentin protein expression in pancreatic tumor tissues from control and anti-Gas6 treated mice (Figures 4C,D). We found that blockade of Gas6 partially reduces vimentin protein expression in cancer cells, although this decrease was not statistically significant.

Pancreatic tumors are usually poorly vascularized but since Gas6 signaling can support endothelial cells proliferation and vascularization (33, 39, 40) we next evaluated whether anti-Gas6 therapy could affect angiogenesis in pancreatic tumors. Pancreatic tumor tissues from control and anti-Gas6 treated mice were stained with the endothelial marker CD31. Whole tumor tissues were scanned and quantified for CD31 expression which remained unchanged in both treatment groups (Supplementary Figures 3A,B). Fourcot et al. (31) showed, in a liver fibrosis model, that Gas6 is secreted by macrophages and fibroblasts and that Gas6 deficiency decreases TGFb and collagen I production by hepatic fibroblasts. Gas6 also stimulates the proliferation of cardiac fibroblasts (41). Since fibrosis and collagen deposition have been suggested to re-strain the metastatic spreading of pancreatic cancer cells (42–45), we next investigated whether Gas6 blockade could affect fibroblasts and collagen deposition in pancreatic tumors. Pancreatic tumor tissues from control and anti-Gas6 treated mice were stained with picrosirius red to assess collagen deposition (Supplementary Figures 3C,D) and for αSMA+ cells (Supplementary Figures 3E,F). Whole tumor tissues were scanned and quantified for collagen deposition (Sirius red positive areas) and αSMA+ cells. We observed a slight increase in collagen deposition in tumors from mice treated with anti-Gas6 antibody compared to control but this increase was not statistically significant (Supplementary Figures 3C,D). αSMA levels remained the same in both treatment groups (Supplementary Figures 3E,F). These findings suggest that the anti-metastatic effect of Gas6 blockade in pancreatic cancer is not due to changes in angiogenesis or fibrosis.

### Gas6 Blockade Does Not Affect Myeloid Cells or T Cells Populations at the Primary Tumor Site, in Peripheral Blood or at the Metastatic Site

**Figure 5:**
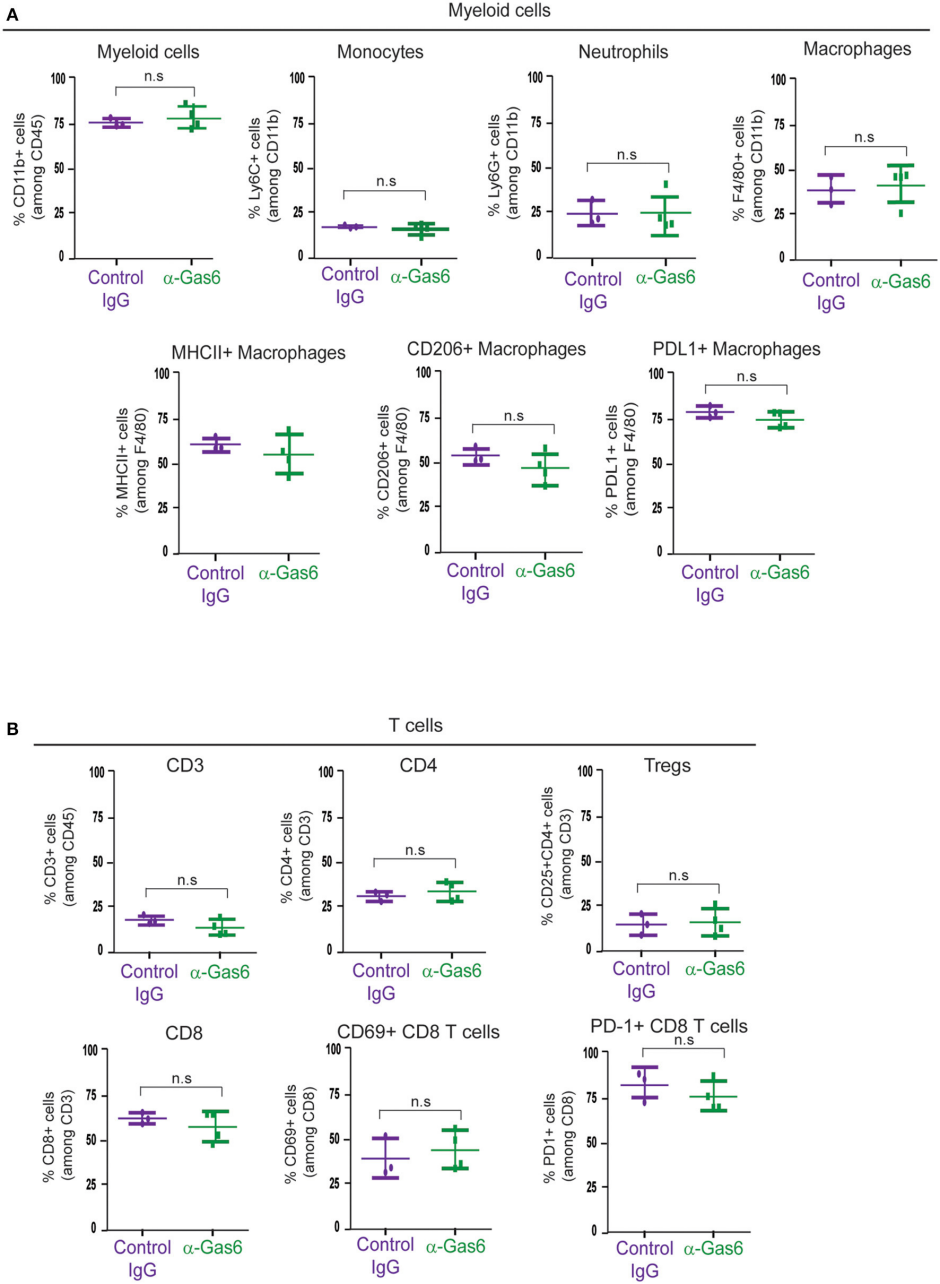
Gas6 blockade does not affect the composition or activation status of myeloid cells and T cells in pancreatic tumors. **(A)** Mass cytometry quantification of CD11b + myeloid cells, Ly6C high/Ly6C low monocytes/MDSCs, Ly6G high/Ly6C low neutrophils/MDSCs, F4/80+ macrophages, MHCII+ macrophages, CD206+ macrophages, and PD-L1+ macrophages in mouse pancreatic tumors treated with control IgG (*n* = 3) or anti-Gas6 neutralizing antibody (*n* = 4). Values shown are mean and SEM. n.s. no statistically significant differences, using unpaired student *T*-test. **(B)** Mass cytometry quantification of CD3+ T cells, CD4+ T cells, CD4+/CD25+ regulatory T cells (Tregs), CD8+ T cells, CD69+/CD8+ T cells and PD-1+/CD8+ T cells in mouse pancreatic tumors treated with control IgG (*n* = 3) or anti-Gas6 neutralizing antibody (*n* = 4). Values shown are mean and SEM. n.s. no statistically significant differences, using unpaired student *T*-test. Graphs were generated with ViSNE data using Cytobank software.

TAM receptors are also expressed by immune cells and regulate myeloid cell and T-cell functions (3, 46). Thus, next, with the aim to understand the systemic effect of Gas6 blockade in myeloid cells and T cells in pancreatic cancer, we evaluated the number and activation status of myeloid cells and T cells in pancreatic tumors, blood and metastatic tissues using mass and flow cytometry. Mass cytometry analysis of myeloid (CD11b+) cells, neutrophils/MDSCs (CD11b+/Ly6G+), monocytes (CD11b+/Ly6C+), macrophages (CD11b+/F4/80+), MHC-II+, CD206+, and PD-L1+ macrophages (Figure 5A) and T cells (CD3+), helper T-cells (CD3+/ CD4+), regulatory T cells (CD3+/CD4+/CD25+), cytotoxic T cells (CD3+/CD8+), activated/exhausted cytotoxic T cells (CD8+/CD69+; CD8+/PD-1+) (Figure 5B) from pancreatic tumors from control vs. anti-Gas6 treated mice did not show any significant differences (Figures 5A,B, Supplementary Figures 4A,B). Similarly, myeloid cell and T cell numbers in blood (Supplementary Figures 5A,B) and metastatic lungs from mice treated with control or anti-Gas6 antibody remained the same (Supplementary Figures 6A,B).

### Gas6 Blockade Restores NK Cell Activation and Infiltration in Metastatic Lesions

**Figure 6:**
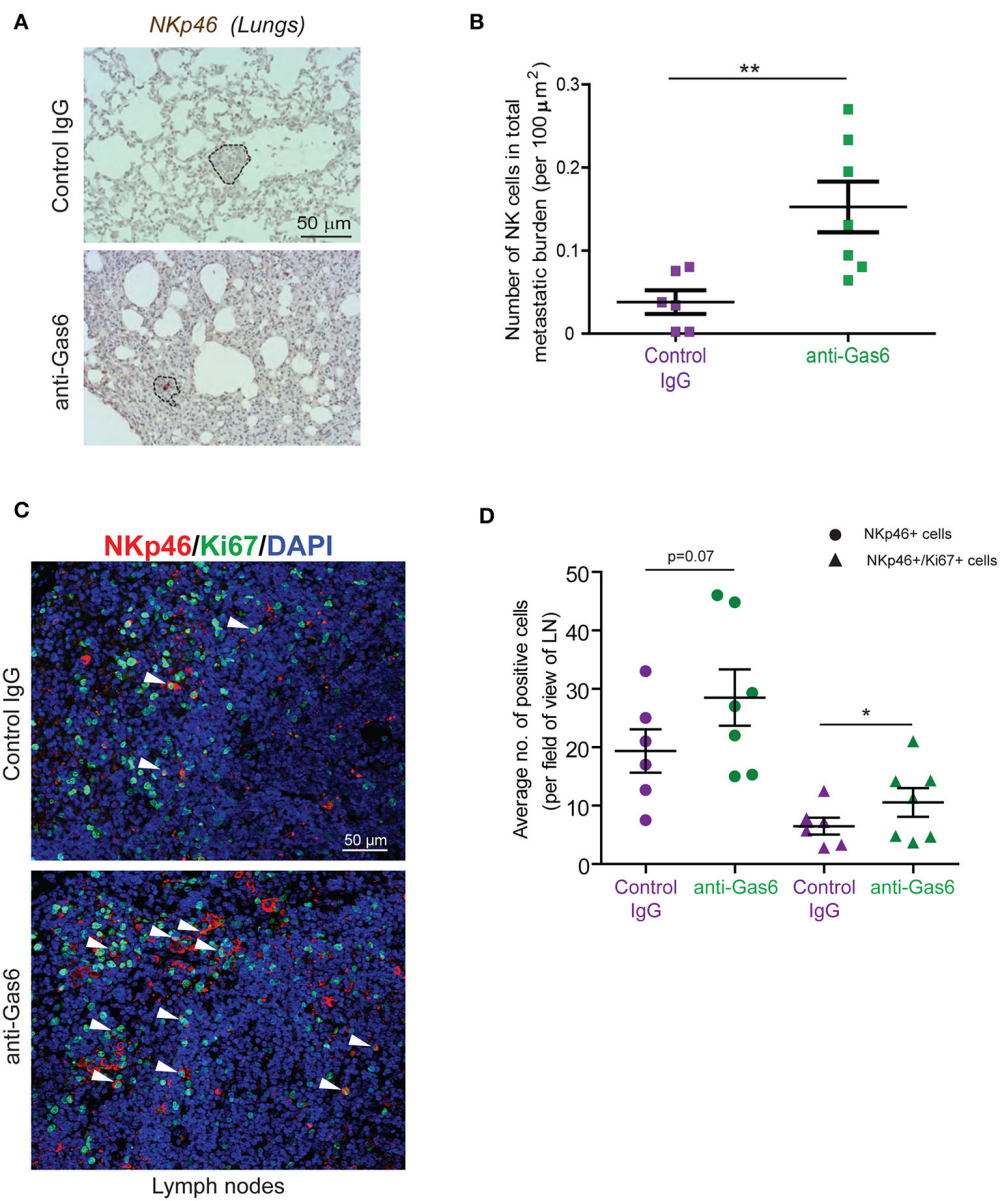
Gas6 blockade increases NK cell numbers in metastatic lungs and in tumor draining lymph nodes. **(A)** Immunohistochemical staining of NK cells in metastatic lungs from pancreatic tumor bearing mice treated with control IgG or anti-Gas6 antibody. Lesions indicated by dashed line and NK cells by red asterisk. Scale bar, 50 μm. **(B)** Quantification of NK cells in metastatic lung tissues from control IgG and anti-Gas6 treated mice. Values shown are the mean and SEM (*n* = 6 mice in IgG treatment group, *n* = 7 mice in anti-Gas6 treatment group). **p ≤ 0.01, using unpaired student *T*-test. **(C)** Immunofluorescent staining of NK cells in mesenteric lymph nodes from pancreatic tumor bearing mice treated with control IgG or anti-Gas6 antibody. NK marker NKp46 is shown in red, Ki67 is shown in green and nuclei were stained with DAPI (in blue). Scale bar, 50 μm. **(D)** Quantification of NK cells in tumor draining lymph nodes from control IgG and anti-Gas6 treated mice. Values shown are the mean and SEM (*n* = 6 mice IgG treatment group and *n* = 7 mice anti-Gas6 treatment group, 3–6 fields/ mouse tissue were quantified). *p ≤ 0.05, using unpaired student *T*-test.

TAM signaling is involved in the development of natural killer (NK) cells (47). In an elegant study, Paolino et al. (9) demonstrated that TAM receptor inhibition activates NK cells cytotoxic function and thereby decreases metastasis in mouse models of breast cancer and melanoma. Thus, we next hypothesized that the anti-metastatic effect of Gas6 blockade we observe in our pancreatic cancer model could be due to a re-activation of NK cells. To test this hypothesis we evaluated NK cells in primary pancreatic tumors, tumor draining lymph nodes, and metastatic lesions of mice treated with control IgG or anti-Gas6 antibody. NK cells were almost absent in all primary tumors from both anti-Gas6 and control treated mice (except for one anti-Gas6 treated pancreatic tumor, Supplementary Figure 7). However, the number of NKp46+ NK cells in lung metastatic lesions was significantly higher in mice treated with anti-Gas6 antibody compared to control treated mice (Figures 6A,B). The number of NK cells, and in particular the number of proliferating NK cells, was also increased in tumor draining lymph nodes from anti-Gas6 treated mice compared to control treated mice (Figures 6C,D).

**Figure 7:**
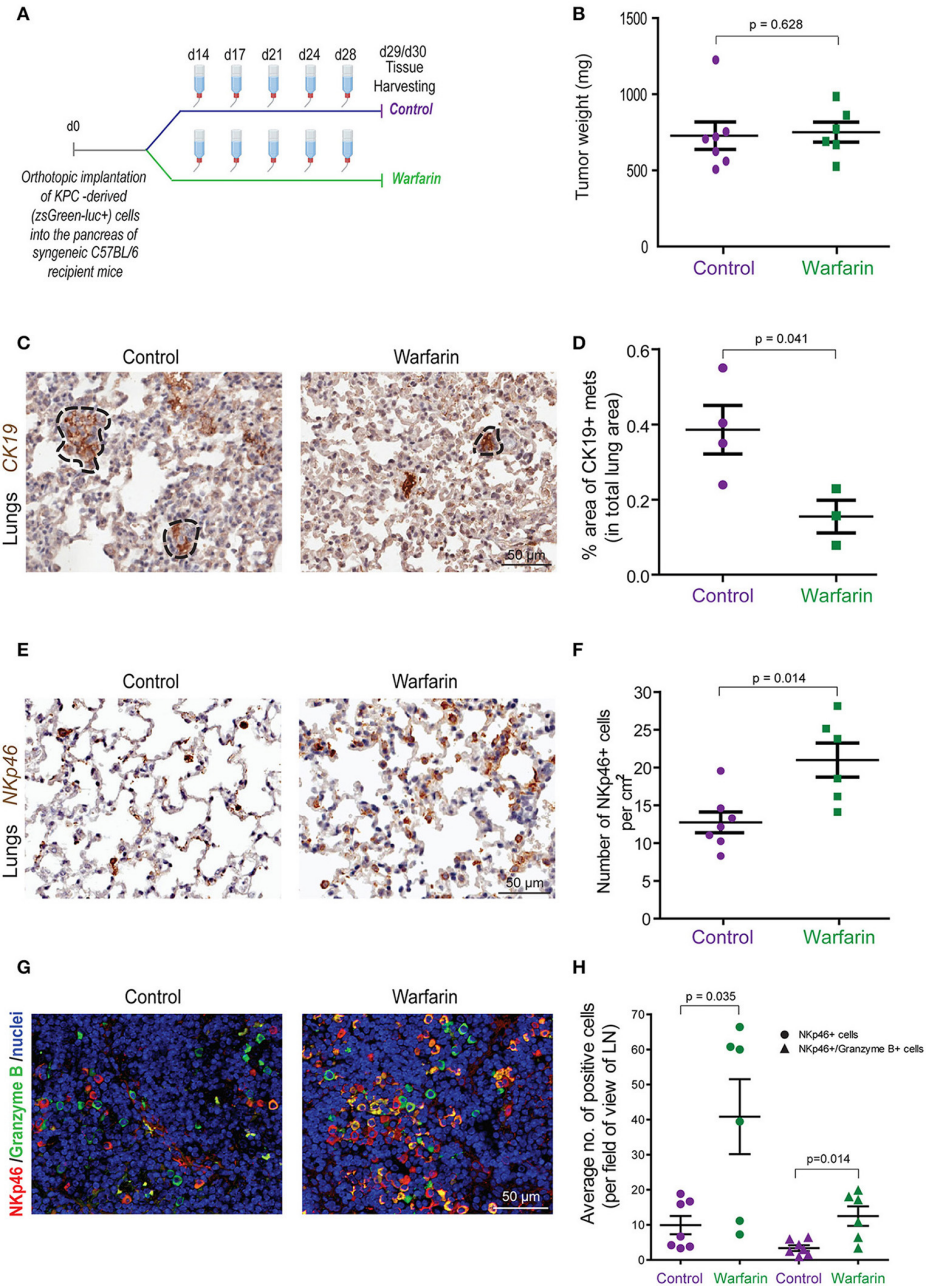
Warfarin decreases pancreatic cancer metastasis and increase NK cell numbers and activation in lymph nodes and at the metastatic site. **(A)** KPC^luc/zsGreen^ (zsGreen) -derived pancreatic tumor cells (FC1242^luc/zsGreen^) were orthotopically implanted into the pancreas of syngeneic C57BL/6 recipient mice. At day 14 the mice were treated with either control drinking water or warfarin sodium in drinking water (0.5 mg/L). Warfarin water was replenished every 3–4 days. Primary tumors, livers, lungs and lymph nodes were harvested at day 29/30. **(B)** Tumor weights from control (*n* = 7) or warfarin (*n* = 6) treated mice. **(C)** Immunohistochemical staining of CK19+ in mice with lung metastases. **(D)** Quantification of the total area of lung metastasis per mouse as a percentage of the total lung area for control (*n* = 4) or warfarin (*n* = 3) treated mice. *p ≤ 0.05 using unpaired student *T*-test. Values shown are mean and SEM. **(E)** Immunohistochemical staining of NKp46+ NK cells in the lungs from pancreatic tumor bearing mice. **(F)** Quantification of the number of NKp46+ NK cells per cm^2^ in the lungs of control (*n* = 7) or warfarin (*n* = 6) treated mice. **(G)** Immunoflourescence staining of NK cells in the mesenteric lymph nodes from pancreatic tumor bearing mice. The NKp46 marker is shown in red, granzyme B is shown in green and the nuclei were stained with DAPI (in blue). Scale bar 50 μm. **(H)** Quantification of the number of NKp46+ and granzyme b+ cells in tumor draining lymph nodes for control (*n* = 7) and warfarin (*n* = 6) treated mice. Values shown are the mean and SEM and 3-10 fields/mouse tissue were quantified.

To further investigate the effect of inhibiting Gas6-Axl signaling in pancreatic cancer progression and metastasis, we performed another *in vivo* experiment, using our syngeneic orthotopic KPC model (described in Figure 1) using warfarin (instead of a neutralizing anti-Gas6 antibody). Warfarin is a vitamin K antagonist that inhibits the vitamin k dependent γ-carboxylation of Gas6 and prevents it from activating TAM receptors (23, 48). Warfarin is currently being tested in pancreatic cancer patients (NCT03536208). Similar to what we observed with the anti-Gas6 treatment, warfarin reduced pancreatic cancer metastasis to the lungs (Figures 7C,D, Supplementary Figures 8A–C) and increased the number and activation of NK cells in lungs (Figures 7E,F) and mesenteric lymph nodes (Figures 7G,H), as shown by the increase in NKp46+ and granzyme B expression. Warfarin treatment also decreased vimentin expression in pancreatic cancer cells, suggesting that warfarin also acts on the cancer cells altering their plasticity (Supplementary Figures 8D,E).

## Discussion

The data presented in this study describe a dual anti-tumor effect of Gas6 blockade in pancreatic tumors, shedding light on the anti-cancer mechanism of action of inhibitors of the Gas6-Axl pathway and supporting the rationale for using anti-Gas6 therapy in pancreatic cancer patients. In these studies we show that blockade of Gas6 in pancreatic tumors, with either an anti-Gas6 neutralizing antibody or with warfarin, acts simultaneously on both the tumor cells, altering their epithelial-mesenchymal phenotype, as well as on NK cells, promoting their activation and recruitment to the metastatic site (Figures 6, 7). These findings suggest that anti-Gas6 therapy decreases pancreatic cancer metastasis by not only affecting cancer cells' plasticity but also by activating NK cells and supporting their tumoricidal function.

So far many studies have focused on the cancer-cell autonomous role of Gas6 and based on their effect on tumor cell proliferation and plasticity several inhibitors of the Gas6-Axl pathways, including warfarin (clinical trial ID: NCT03536208) are currently being tested in pancreatic cancer patients.

**Figure 8:**
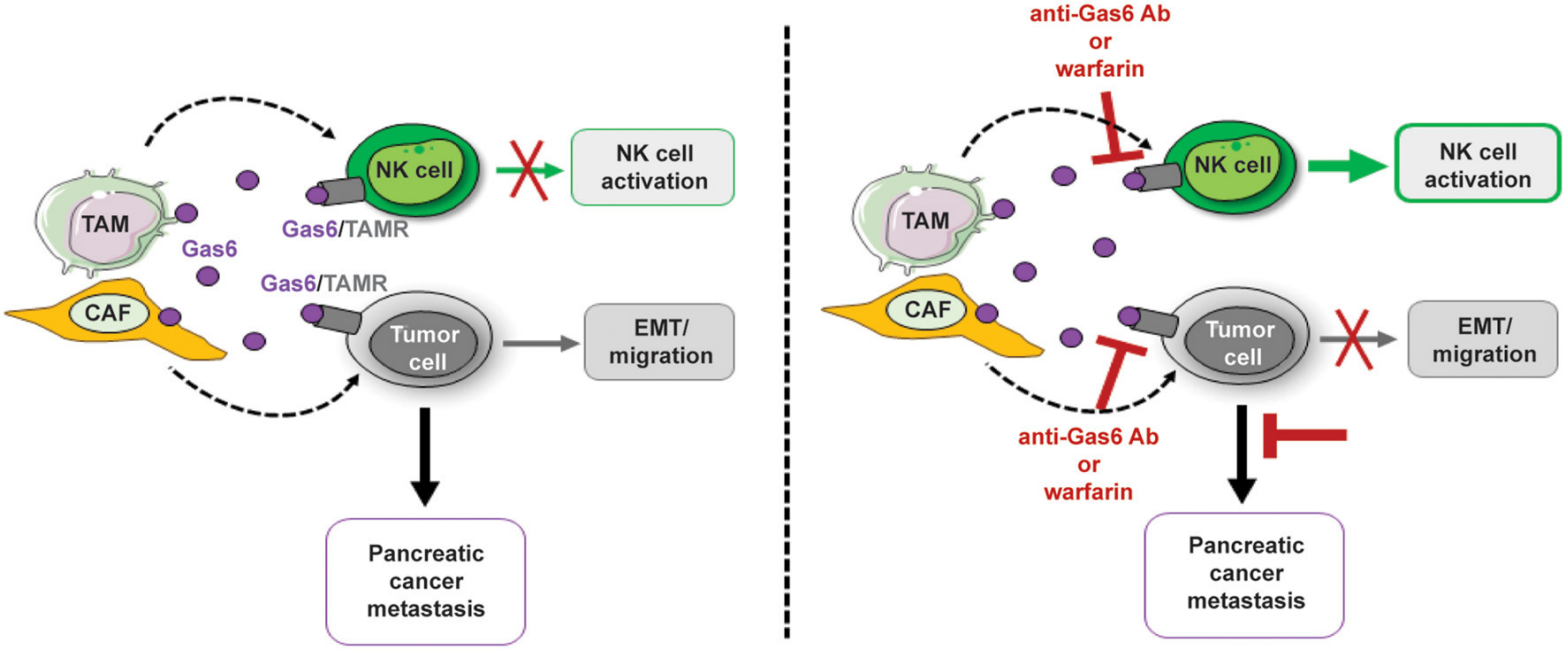
Schematics depicting the multifunctional role of stroma-derived Gas6 in pancreatic cancer. *In vivo* blockade of Gas6 signaling with a neutralizing anti-Gas6 antibody or warfarin, partially reverses tumor cells EMT and activates NK cells, leading to a decrease in pancreatic cancer metastasis.

Our studies show that inhibition of Gas6 signaling in pancreatic cancer not only affects the tumor cells but notably affects the NK cells (Figure 8). Our findings suggest that the activation status of NK cells should also be assessed in cancer patients and could be used as a biomarker to monitor response to anti-Gas6/Axl therapies.

Gas6/Axl signaling is a negative regulator of the immune system and inhibition of the Gas6-Axl signaling leads to autoimmunity (4). While the function of Gas6-Axl signaling on tumor cell proliferation, EMT, migration and drug resistance has been extensively studied (5), only a few studies have investigated the role of Gas6/Axl signaling in the immune system in the context of cancer (7, 9, 24). Guo et al. (7) found that the Axl inhibitor R428 inhibited tumor growth of subcutaneously implanted murine 4T1 breast cancer cells and intra-peritoneally implanted murine ID8 ovarian cancer cells by activating CD4+ and CD8+ T cells. Inspired by this study, we investigated whether, in our pancreatic cancer model, Gas6 blockade supports the activation of T cells. Unlike Guo et al. (7) we did not observe any statistically significant difference in CD4+ or CD8+ T cells in pancreatic tumors, blood or metastatic tissues, in control vs. anti-Gas6 treated mice. Ludwig et al. (24) found that treating mouse pancreatic tumors with the Axl inhibitor BGB324 decreased the number of tumor associated macrophages (TAMs) in some but not all tumor models. In our study, blocking Gas6 did not significantly affect TAMs, other myeloid cell populations or T cells in primary tumors, blood or metastatic organs. These different results observed in these studies may be explained by the differences in the tumor models used (breast cancer vs. pancreatic cancer; xenograft vs. syngeneic models) and the differences in the therapies used (inhibition of AXL receptor vs. inhibition of Gas6 ligand which binds all TAM receptors). In another study, Paolino et al. (9) showed that TAM receptor inhibition activates NK cells in mouse tumor models of melanoma and breast cancer leading to decreased tumor growth. In agreement with these findings, we found that blocking Gas6 in mice bearing pancreatic tumors, increases NK cell number and activation in tumor draining lymph nodes and lungs, and decreases pancreatic cancer metastasis.

Inhibition of the Gas6-Axl pathway has been shown to reverse EMT, tumor migration and intra-tumoral micro-vessel density in pancreatic cancer (23). In agreement with these findings, we found that inhibition of Gas6 signaling decreases the expression of the EMT transcription factors *Snail 1, Snail 2, Zeb2*, and vimentin expression in pancreatic cancer cells. *E-cadherin* and *b-catenin* levels were also decreased upon anti-Gas6 treatment suggesting that blockade of Gas6 signaling leads to a partial MET or hybrid E/M phenotype. Partial EMT is a phenomenon often observed in cancer, where cancer cells that originate from epithelial cells exhibit both mesenchymal and epithelial characteristics. The ability of cancer cells to undergo partial EMT, rather than complete EMT and to maintain the expression of both E-cadherin and vimentin poses a higher metastatic risk (37, 44). Pancreatic tumors are usually hypo-vascularized compared to a normal pancreas and anti-angiogenic therapies have not been successful in pancreatic cancer (49). Similar to the human disease, in our pancreatic mouse tumor model, tumors are poorly vascularized and blocking Gas6 did not show any further decrease in tumor vascularization. Loges et al. (11) previously showed that tumor associated macrophages (TAMs) produce Gas6 in various mouse tumor models. In our study we found that both TAMs and CAFs are the main sources of Gas6 in pancreatic tumors. These findings suggest that the abundance of TAMs and CAFs in pancreatic cancer patients could be used to determine which patients would benefit the most from anti-Gas6 therapy.

In conclusion, our studies suggest that in pancreatic cancer, Gas6 is secreted by both TAMs and CAFs and blockade of Gas6 signaling has a dual anti-metastatic effect by acting on both the tumor cells and the NK cells. Thus, inactivation of Gas6 signaling can promote anti-tumor immunity, via NK cell activation, in pancreatic tumors. Since this Gas6-dependent immune regulation of NK cells is also conserved in humans, anti-Gas6-Axl therapies are likely to promote anti-tumor immunity, via NK cell activation, in pancreatic cancer patients. This study provides further mechanistic insights into the mode of action of anti-Gas6 therapies and suggests the use of NK cells as an additional biomarker for response to anti-Gas6 therapies in pancreatic cancer patients.

## Materials and Methods

### Generation of Primary KPC-Derived Pancreatic Cancer Cells

The murine pancreatic cancer cells KPC FC1242 were generated in the Tuveson lab (Cold Spring Harbor Laboratory, New York, USA) isolated from PDA tumor tissues obtained from LSL-Kras^G12D^; LSL-Trp53^R172H^; Pdx1-Cre mice of a pure C57BL/6 background as described previously with minor modifications (50).

### Generation of Primary Macrophages, Primary Pancreatic Fibroblasts, Macrophage (MCM, and Fibroblasts (FCM) Conditioned Media

Primary murine macrophages were generated by flushing the bone marrow from the femur and tibia of 6–8 week-old C57BL/6 mice followed by incubation for 5 days in DMEM containing 10% FBS and 10 ng/mL murine M-CSF (Peprotech). Primary pancreatic stellate cells were isolated from the pancreas of C57BL/6 mice by density gradient centrifugation, and were cultured on uncoated plastic dishes in IMDM with 10% FBS and 4 mM L-glutamine. Under these culture conditions pancreatic stellate cells activated into myofibroblasts.

To generate macrophage and fibroblast conditioned media, cells were cultured in serum free media for 24–36 h, supernatant was harvested, filtered with 0.45 μm filter, concentrated using StrataClean Resin (Agilent Technologies) and immunoblotted for Gas6 (R&D Systems, AF885).

### Immunoblotting

FC1242 cells were plated in DMEM media with 10% FBS for 24 h, harvested and lysed in RIPA buffer (150 mM NaCl, 10 mM Tris-HCl pH 7.2, 0.1% SDS, 1% Triton X-100, 5 mM EDTA) supplemented with a complete protease inhibitor mixture (SIGMA), a phosphatase inhibitor cocktail (Invitrogen), 1 mM PMSF and 0.2 mM Na_3_VO_4_. Immunoblotting analyses was performed using phospho-Axl antibody (R&D systems, AF2228).

### Syngeneic Orthotopic Pancreatic Cancer Model

1 × 10^6^ primary KPC^luc/zsGreen^ cells (FC1242^luc/zsGreen^) isolated from a pure C57Bl/6 background were implanted into the pancreas of immune-competent syngeneic C57Bl/6 six- to 8 week-old female mice, and tumors were established for 2 weeks before beginning treatment. Mice were administered i.p. with Gas6 neutralizing antibody (R&D systems, AB885) (2 mg/kg), or IgG isotype control antibody, every 3–4 days or warfarin sodium in drinking water (0.5 mg/L) which was replenished every 3–4 days, for 15 days before harvest.

### Analysis and Quantification of Immune Cells in Pancreatic Tumors by Mass Cytometry

Pancreatic tumors were resected from the mice and mechanically and enzymatically digested in Hanks Balanced Salt Solution (HBSS) with 1 mg/mL Collagenase P (Roche) Cell suspensions were centrifuged for 5 min at 1,500 rpm, resuspended in HBSS and filtered through a 500 μm polypropylene mesh (Spectrum Laboratories). Cells were resuspended in 1 mL 0.05%Trypsin and incubated at 37°C for 5 min. Cells were filtered through a 70 μm cell strainer and resuspended in Maxpar cell staining buffer (Fluidigm). The samples were centrifuged for 5 min at 450 *x g* and supernatant removed. The cells were subsequently stained with Cell-ID 195-Cisplatin (Fluidigm) viability marker diluted 1:40 in Maxpar PBS (Fluidigm) for 5 min. Cells were centrifuged at 450 *x g* for 5 min and washed twice in Maxpar cell staining buffer. Samples were blocked for 10 min on ice with 1:100 diluted FC Block (BD Pharmingen, Clone 2.4G2) and metal-conjugated antibody cocktail added and incubated for 30 min at 4°C. Antibodies were used at the concentrations recommended by manufacturers. Cells were washed twice in cell staining buffer and stained with 125 μM 191-Intercalator-Ir (Fluidigm) diluted in 1:2,000 Maxpar fix and perm buffer (Fluidigm) overnight at 4°C. The cells were washed twice in Maxpar cell staining buffer and centrifuged at 800 *x g* for 5 min. A post-fix was performed by incubating the cells in 1.6% PFA for 30 min at RT. Cells were washed twice in 18Ω distilled water (Fluidigm), mixed 1:10 with EQTM Four Element Calibration Beads (Fluidigm) and acquired on the Helios CyTOF system (Fluidigm). Samples were acquired at a rate of around 200 cells/s. All generated FCS files were normalized and beads removed (51). All analysis was performed in Cytobank: Manual gating was used to remove dead cells (195Pt+) and debris and to identify single cells (191 Ir+).

viSNE analysis was performed on the data utilizing t-stochastic neighbor embedding (t-SNE) mapping based on high dimensional relationships. CD45+ population selected by manual gating was used as the starting cell population and using proportional sampling viSNE unsupervised clustering was performed. Manual gating was then performed on the viSNE map created to determine cell population percentages. Spanning-tree Progression Analysis of Density-normalized Events (SPADE) analysis was performed in Cytobank using manually gated CD45+ cells, 200 target number of nodes and 10% down sampled events, to equalize the density in different parts of the cloud. In Cytobank SPADE analysis edge number between nodes indicates levels of similarity, with more steps indicating less similarity across channels used to create the tree. Node localization and edge length cannot be used to infer similarity in this analysis. Event number is indicated by both color scale and node size (which is proportional to the number of cells present in each cluster). Gating of cell populations was performed to identify major cell populations and percentages.

### FACS Sorting and Analysis of Blood and Lungs by Flow Cytometry

Single cell suspensions from murine primary pancreatic tumors and pulmonary metastasis were prepared by mechanical and enzymatic disruption and tumor cells, tumor associated macrophages and stromal cells were analyzed and sorted using flow cytometry (FACS ARIA II, BD Bioscience). Samples were digested as outlined above, the cells were then filtered through a 70 μm cell strainer and resuspended in PBS + 1% BSA, blocked for 10 min on ice with FC Block (BD Pharmingen, Clone 2.4G2) and stained with Sytox® blue viability marker (Life Technologies) and conjugated antibodies anti-CD45-PE/Cy7 (Biolegend, clone 30-F11) and anti-F4/80-APC (Biolegend, clone BM8).

Blood was collected from mice via tail vein bleed in EDTA-tubes. Red blood cell lysis was performed and resulting leukocytes were resuspended in PBS + 1% BSA and blocked for 10 min on ice with FC block and stained with Sytox® blue viability marker and conjugated antibodies anti-CD45-APC/Cy7 (Biolegend, 103115), anti-CD11b-APC (Biolegend, 101212), anti-Ly6G-PerCP-Cy5.5 (Biolegend, 127616), anti-Ly6C-PE (Biolegend, 128008), anti-CD3-PE-Cy7 (Biolegend, 100320), anti-CD4-PE (Biolegend, 100408), and anti-CD8-PerCP-Cy5.5 (Biolegend, 100734). Cell analysis was performed using FACS Canto II.

### Gene Expression

Total RNA was isolated from FACS sorted tumor cells, tumor associated macrophages and non-immune stromal cells from primary pancreatic tumors as described in Qiagen Rneasy protocol. Total RNA from the different cell populations was extracted using a high salt lysis buffer (Guanidine thiocynate 5 M, sodium citrate 2.5 uM, lauryl sarcosine 0.5% in H_2_O) to improve RNA quality followed by purification using Qiagen Rneasy protocol. cDNA was prepared from 1 μg RNA/sample, and qPCR was performed using gene specific QuantiTect Primer Assay primers from Qiagen. Relative expression levels were normalized to *gapdh* expression according to the formula <2∧– (Ct *gene of interest*—Ct *gapdh*) (52).

### Quantification of Metastasis

#### By IVIS Imaging

IVIS spectral imaging of bioluminescence was used for orthotopically implanted tumor cells expressing firefly luciferase using IVIS spectrum system (Caliper Life Sciences). Organs were resected for *ex vivo* imaging coated in 100 μL D-luciferin (Perkin Elmer) for 1 min and imaged for 2 min at automated optimal exposure. Analysis was performed on the Living Image software (PerkinElmer) to calculate the relative bioluminescence signal from photon per second mode normalized to imaging area (total flux) as recommended by the manufacturer.

#### By H&E Staining

FFPE lungs were serially sectioned through the entire lung using microtome at 4 μm thickness. Sections were stained with H&E and images were taken using a Zeiss Observer Z1 Microscope (Zeiss) to identify metastatic foci. The number of foci were counted, and the total area of metastatic foci was measured using Zen imaging software. Metastatic burden was calculated by the following equations:

No. of foci per 100 mm^2^: *(Average no. foci per section/ average tissue area per section (mm*^2^*)* ^*^100

Average metastatic lesion size (mm^2^): *Average total area of metastasis (mm*^2^*)/ average number of foci per section*

Total metastatic burden: *Sum of area of each foci of each section*.

#### By CK19 Staining

FFPE Lung tissue sections were also stained for cytokeratin 19 (CK19). The slides were scanned with an Aperio slide scanner and the whole lung tissue was quantified for CK19 expression using Image J.

### Immunohistochemistry and Immunofluorescence

Deparaffinization and antigen retrieval was performed using an automated DAKO PT-link. Paraffin-embedded pancreatic tumors, lymph nodes, and lung metastasis tissues were immuno-stained using the DAKO envision+ system-HRP.

#### Antibodies and Procedure Used for Immunohistochemistry

All primary antibodies were incubated for 2 h at room temperature: αSMA (Abcam, ab5694 used at 1:200 after low pH antigen retrieval), CD31 (Cell signaling technology, CST 77699 used at 1:100 after low pH antigen retrieval), NKp46 (Biorbyt, orb13333 used at 1:200) and AF2225 (used at 1:50 after low pH antigen retrieval), CK19 (ab53119 used at 1:100 after low pH antigen retrieval), and CD68 (Abcam, ab31630 used at 1:400 after low pH antigen retrieval). Subsequently, samples were incubated with secondary HRP-conjugated antibody (from DAKO envision kit) for 30 min at room temperature. All antibodies were prepared in antibody diluent from Dako envision kit. Staining was developed using diamino-benzidine and counterstained with hematoxylin.

Human paraffin-embedded PDA tissue sections were incubated overnight at RT with the following primary antibodies: phospho-Axl (R&D, AF2228, used 1:500 after high pH antigen retrieval), CD163 (Abcam, ab74604 pre-diluted after low pH antigen retrieval), αSMA (Abcam, ab5694 used 1:100 after low pH antigen retrieval).

#### Antibodies and Procedure Used for Immunofluorescence

After low pH antigen retrieval, lymph node tissue sections derived from mice bearing pancreatic tumors were incubated overnight at RT with the following primary antibodies: NKp46 (R&D systems AF2225, used at 1:25), Ki67 (Abcam ab15580, used at 1:1000), vimentin (Abcam ab92547, used at 1:400), and Granzyme B (ab4059, used at 1:600). Vimentin expression was quantified on cancer cells located at the edge of pancreatic tumors. Samples were washed with PBS and incubated with donkey anti-goat 594 (Abcam ab150132) and donkey anti-rabbit 488 (Abcam ab98473) secondary antibodies, respectively, all used at 1:300 and DAPI at 1:600 for 2 h at RT. Slides were washed with PBS, final quick wash with distilled water and mounted using DAKO fluorescent mounting media.

After low pH antigen retrieval, mouse tissue sections derived from paraffin embedded pancreatic tumors were incubated with vimentin (ab92547, used at 1:400) overnight at 4c. Goat anti-rabbit 594 (ab150080) secondary was used at 1:300 and DAPI at 1:600 for 2 h at RT.

Human PDA frozen tissue sections were fixed with cold acetone, permeabilized in 0.1% Triton, blocked in 8% goat serum and incubated overnight at 4°C with anti-phospho-Axl (R&D, AF2228, diluted 1:200) CK11 (Cell signaling, CST 4545, diluted 1:200), followed by fluorescently labeled secondary antibodies goat anti mouse 488 (Abcam ab98637), goat anti-rabbit 594 (Abcam ab98473) used at 1:300 for 2 h at RT slides were washed with PBS, final quick wash with distilled water and mounted using DAKO fluorescent mounting media.

### Picrosirius Red Staining

FFPE PDA tumor sections were deparaffinized in two 5 min xylene washes and through decreasing alcohol washes of 100, 75, and 65% each 5 min. The slides were washed for 5 min in distilled water and incubated in 0.2% phosphomolybdic acid for 5 min. After washing in PBS, were stained with 0.1% Sirius red F3B in saturated picric acid solution for 90 min. After two rinses in acidified water the slides were stained with fast green (0.01%) for 1 min. The sections were rinsed twice in acidified water were rapidly dehydrated using three steps of 100% ethanol and two xylene incubations of 30 s.

### Statistical Methods

Statistical significance for *in vitro* assays and animal studies was assessed using unpaired two-tailed Student *t*-test and the GraphPad Prism 5 program. All error bars indicate SD for *in vitro* studies and SEM for animal studies.

### Institutional Approvals

All studies involving human tissues were approved by the University of Liverpool and were considered exempt according to national guidelines. Human pancreatic cancer samples were obtained from the Liverpool Tissue Bank from patients that consented to use the surplus material for research purposes. All animal experiments were performed in accordance with current UK legislation under an approved project license (reference number: 403725). Mice were housed under specific pathogen-free conditions at the Biomedical Science Unit at the University of Liverpool.

## Data Availability Statement

All datasets generated for this study are included in the article/Supplementary Material.

## Ethics Statement

The studies involving human participants were reviewed and approved by the University of Liverpool and were considered exempt according to national guidelines. Human pancreatic cancer samples were obtained from the Liverpool Tissue Bank from patients that consented to use the surplus material for research purposes. The patients/participants provided their written informed consent to participate in this study. All animal experiments were performed in accordance with current UK legislation under an approved project license (reference number: 403725). Mice were housed under specific pathogen-free conditions at the Biomedical Science Unit at the University of Liverpool.

## Author Contributions

LI designed experiments and performed most of the experiments including *in vivo* experiments, mass cytometry/flow cytometry, cell isolations, immunohistochemical stainings, and qPCR experiments. TL designed and performed qPCR experiments, tissue stainings, and *in vivo* experiment with warfarin treatment. AM designed experiments, helped with tissue harvesting and tissue stainings, conceived and supervised the project. MS provided conceptual advice and help with *in vivo* experiments. AM and LI wrote the manuscript. All authors helped with the analysis and interpretation of the data, the preparation of the manuscript, and approved the manuscript.

### Conflict of Interest

The authors declare that the research was conducted in the absence of any commercial or financial relationships that could be construed as a potential conflict of interest.
