# Supplementary material for: Blockade of Stromal Gas6 Alters Cancer Cell Plasticity, Activates NK Cells, and Inhibits Pancreatic Cancer Metastasis
Source: Front Immunol. 2020 Feb 27;11:297. doi: 10.3389/fimmu.2020.00297 (PMC7056881; doi:10.3389/fimmu.2020.00297)
Supplement: Supplementary file 1 [file Data_Sheet_1.docx]

**Supplementary Figure legends**

**Supplementary Figure 1. Anti-Gas6 treatment reduces metastasis.**

**(A)** Immunohistochemical staining of phospho-AXL in normal pancreas tissue, pancreatic tumors treated with IgG (control) or anti-Gas6 antibody. Scale bars 100 μm**. (B)** Immunoblotting of FC1242zsGreen tumour cells for phospho-AXL expression, detected, as expected at 140kDa. **(C)** Metastatic incidence, measured by bioluminescent signalling (IVIS imaging technology) in lungs, livers and lymph nodes of control and anti-gas6 treated mice. **(D)** Quantification of metastasis in lungs, livers, and mesenteric lymph nodes, by *ex-vivo* bioluminescent signalling (IVIS imaging technology) **(E)** Immunohistochemical staining of CK19 in lung metastasis from mice treated with IgG (control) or anti-Gas6 antibody. Scale bar 50 µm. **(F)** Quantification of number of lung metastatic foci per mouse treated with control IgG or anti-Gas6 antibody identified by CK19 staining. *** p ≤ 0.001, using unpaired student T test, error bars represent SEM (n=6 IgG treatment group, n= 7 anti-Gas6 treatment group). **(G)** Average size of pulmonary metastatic lesions in mice treated with control IgG or anti-Gas6 antibody identified by CK19 staining. ** p ≤ 0.01, using unpaired student T test, error bars represent SEM (n=6 IgG treatment group, n=7 anti-Gas6 treatment group).

**Supplementary Figure 2. Gating strategy used to FACS-sort tumor cells, tumor associated macrophages and stromal cells from PDA tumors.**

**(A**) FACS gating strategy of murine orthotopic PDA tumours for tumor cells (Sytox-, CD45-, zsGreen+), non-immune stromal cells (Sytox-, CD45-, zsGreen-), M1-like macrophages (Sytox-, CD45+, F4/80+, CD206-) and M2-like macrophages (Sytox-, CD45+, F4/80+, CD206+). **(B)** qPCR analysis of *αSMA* expression relative to GAPDH in FACS sorted M1-like and M2-like macrophages, stromal cells and tumor cells. Values shown are the mean and SD (n=3).

**Supplementary Figure 3. Gas 6 blockade in pancreatic tumors does not affect angiogenesis or collagen deposition.**

**(A)** Images of whole scanned pancreatic tumors from control and anti-Gas6 treated mice stained for CD31. **(B)** Quantification of CD31+ staining in total tumor area. Values shown are the mean and SEM (n=3 per treatment group). n.s. no statistically significant differences, using unpaired student T test**. (C)** Images of whole scanned pancreatic tumors from control and anti-Gas6 treated mice stained with picrosirius red. **(D)** Quantification of picrosirius red+ staining in total tumor area. Values shown are the mean and SEM (n=7 per treatment group). n.s. no statistically significant differences, using unpaired student T test**. (E)** Images of whole scanned pancreatic tumors from control and anti-Gas6 treated mice stained with αSMA. **(F)** Quantification of αSMA + staining in total tumor area. Values shown are the mean and SEM (n=7 per treatment group). n.s. no statistically significant differences, using unpaired student T test**.**

**Supplementary Figure 4. SPADE analysis of CyTOF analysed pancreatic tumors.**

**(A)**  Table of heavy metal-conjugated antibodies (from Fluidigm) used for CyTOF analysis. **(B)** Control IgG treated and anti-Gas6 treated mice SPADE tree figures of CD45+ immune cells. Manual gating identified myeloid cells (MHCII+, CD11b+) monocytes (Ly6C high/Ly6G low), neutrophils (Ly6C low/Ly6G high), M1-like macrophages (F4/80+, CD68+), M2-like macrophages (F4/80+, CD206+) and T cells (CD3+): T helper (CD4+), Cytolytic T cells (CD8+) and T regulatory cells (CD25+). Key for each SPADE tree indicates node size and color, and are both representative of cell number per node.

**Supplementary Figure 5. Anti-Gas6 treatment does not alter myeloid cell or T cell in the peripheral blood.**

**(A)** FACS analysis of control IgG and anti-Gas6 treated mice peripheral blood (n=8 per treatment group) for the presence of myeloid cells (CD11b+), monocytes (Ly6C high/Ly6G low) and neutrophils (Ly6C low/Ly6G high). Values shown are the mean and SEM (n=8). n.s. no statistically significant differences, using unpaired student T test**. (B)** T cell populations: pan-T cell (CD3+), T helper cells (CD4+) and cytotoxic T cells (CD8+). Values shown are the mean and SEM (n=8). n.s. no statistically significant differences, using unpaired student T test**.**

**Supplementary Figure 6. Anti-Gas6 treatment does not alter myeloid cell or T cell levels in the lungs**

**(A)** FACS analysis of Control IgG and anti-Gas6 treated mice lung metastasis (n=5 per treatment group) for presence of myeloid cells (CD11b+), monocytes (Ly6C high/Ly6G low+) and neutrophils (Ly6C low/Ly6G high+). Values shown are the mean and SEM (n=4). n.s. no statistically significant differences, using unpaired student T test**. (B)** T cell populations: pan-T cell (CD3+), T helper cells (CD4+) and cytotoxic T cells (CD8+). Values shown are the mean and SEM (n=4). n.s. no statistically significant differences, using unpaired student T test**.** error bars show SD (n=4).

**Supplementary Figure 7. Anti-Gas6 treatment does not increase NK cell numbers in primary pancreatic tumors.**

Representative immunohistochemical staining of NK cells in primary pancreatic tumors from mice treated with control IgG or anti-Gas6 antibody (n=6). Scale bar, 100 μm. Black arrows indicate NK cells.

**Supplementary Figure 8. Warfarin treatment reduces metastasis and reduces vimentin expression in pancreatic tumours**

**(A)** Immunohistochemical staining of phospho-AXL in pancreatic tumors from mice treated with normal water (control) or warfarin in drinking water **(B)** Metastatic incidence, measured by bioluminescent signalling (IVIS) in lungs, livers and lymph nodes of control and warfarin treated mice. **(C)** Quantification of metastasis to the lungs, liver and lymph nodes in by bioluminescent imaging (IVIS). **(D)** Representative immunofluorescent images of vimentin staining at the periphery of pancreatic tumors from control and warfarin treated mice. The dashed lines highlight the areas quantified. **(E)** Quantification of vimentin protein expression levels in pancreatic tumors. Data are displayed as mean and SEM and represent 5 images per mouse, with 7 animals per treatment group. * p ≤0.05 using unpaired student T test.
