# Supplementary figures and images for: Blockade of Stromal Gas6 Alters Cancer Cell Plasticity, Activates NK Cells, and Inhibits Pancreatic Cancer Metastasis
Source: Front Immunol. 2020 Feb 27;11:297. doi: 10.3389/fimmu.2020.00297 (PMC7056881; doi:10.3389/fimmu.2020.00297)

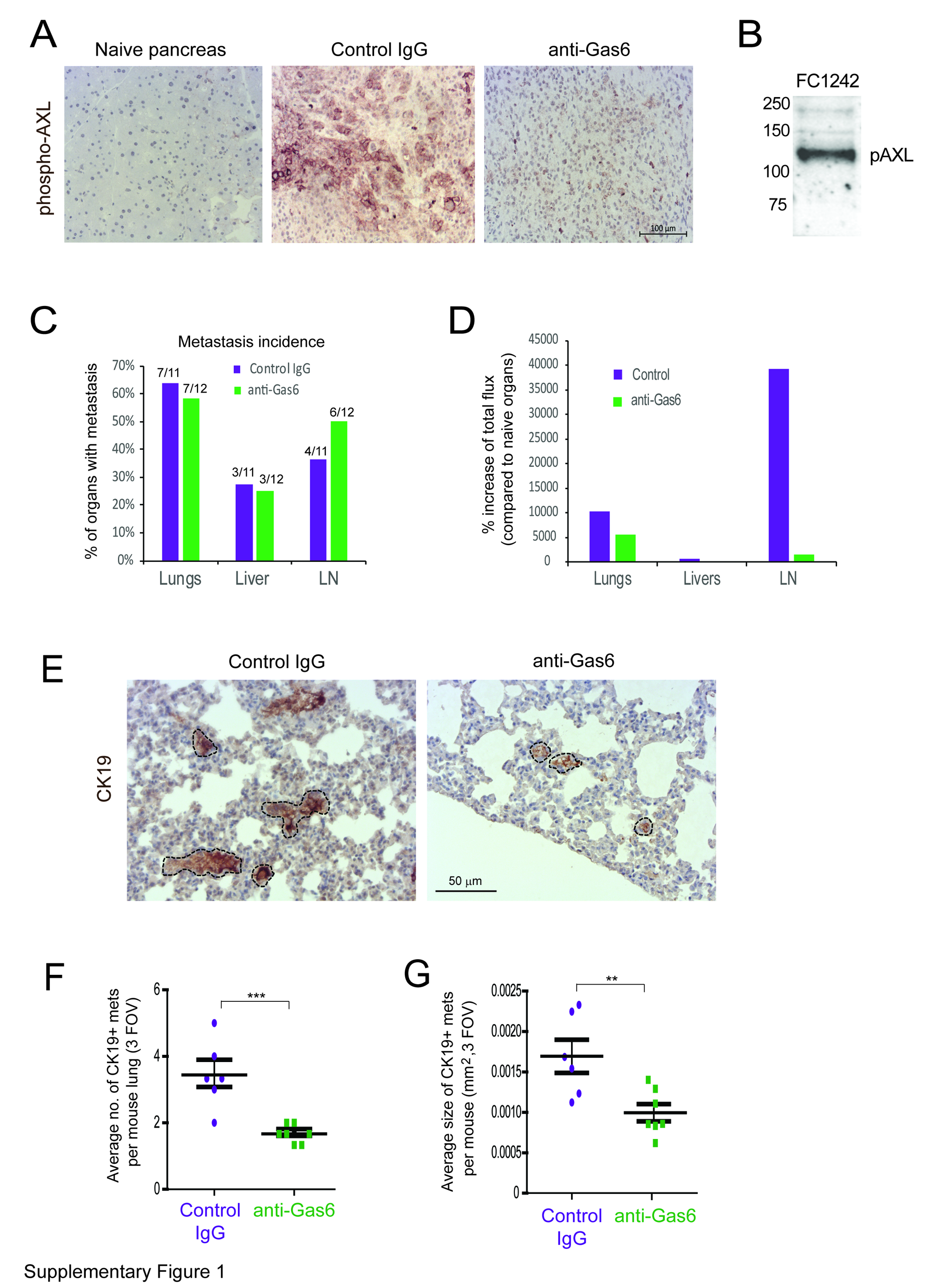

Supplement: Supplementary file 2 [file Image_1.TIF]

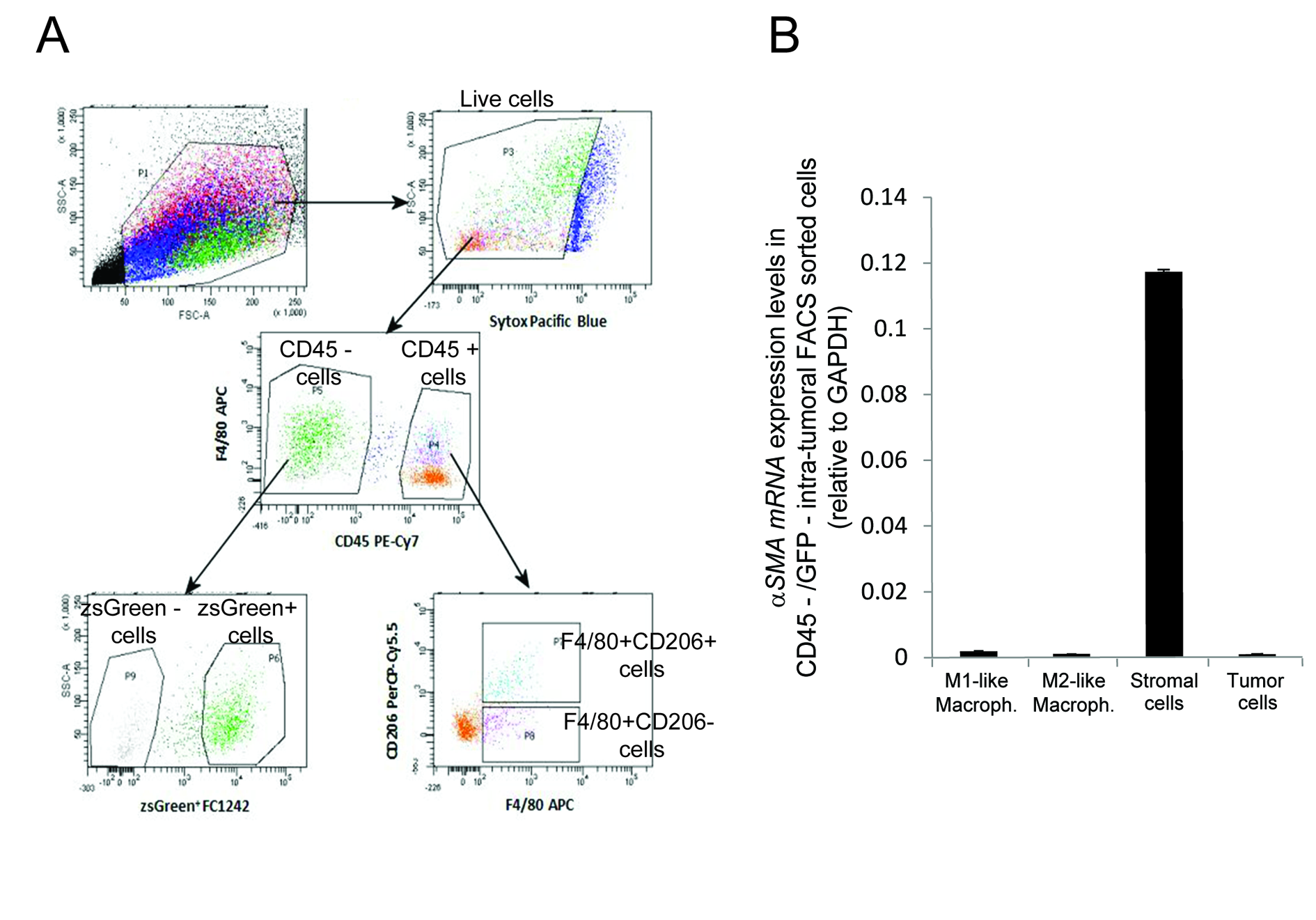

Supplement: Supplementary file 3 [file Image_2.TIF]

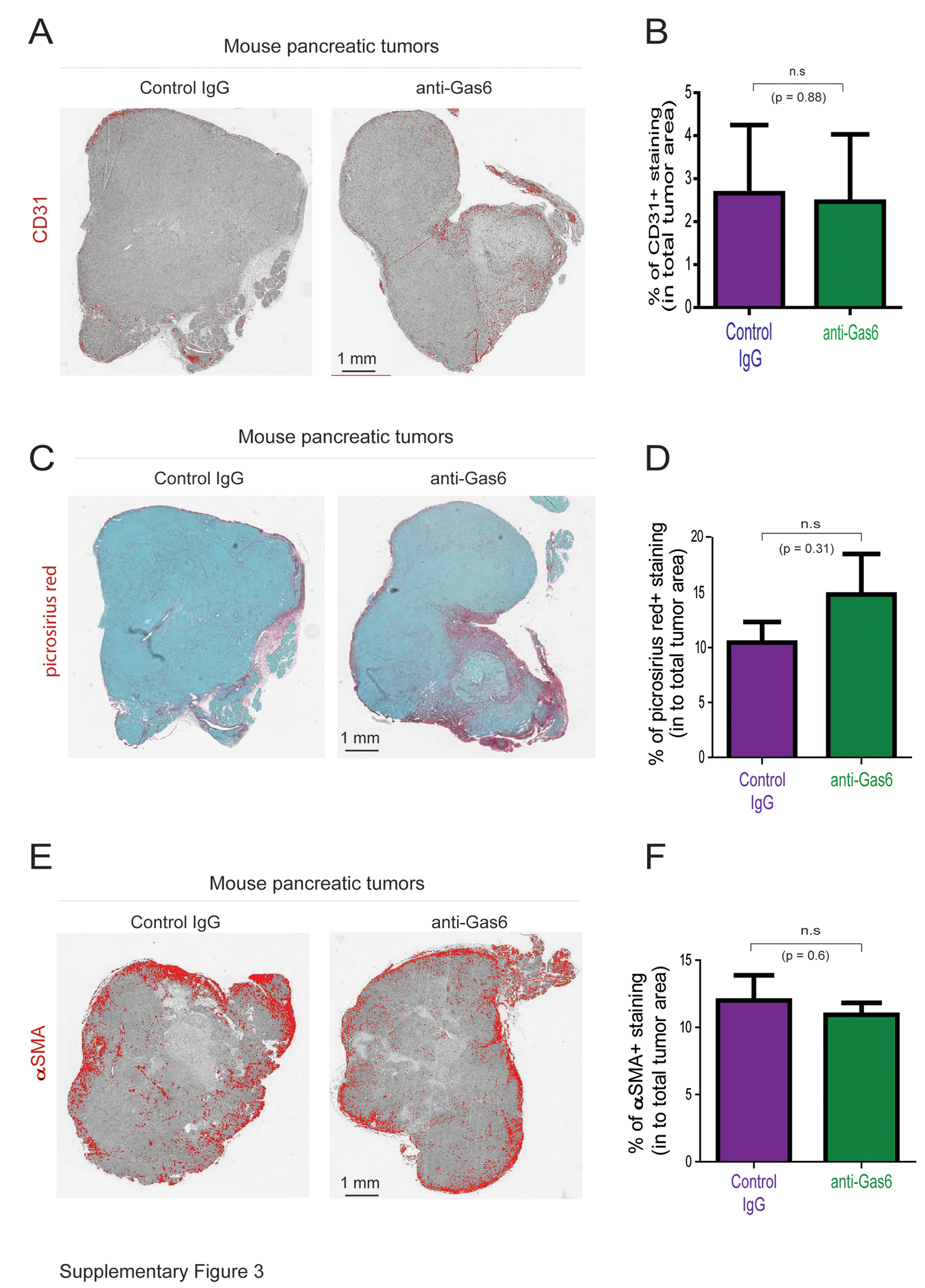

Supplement: Supplementary file 4 [file Image_3.TIF]

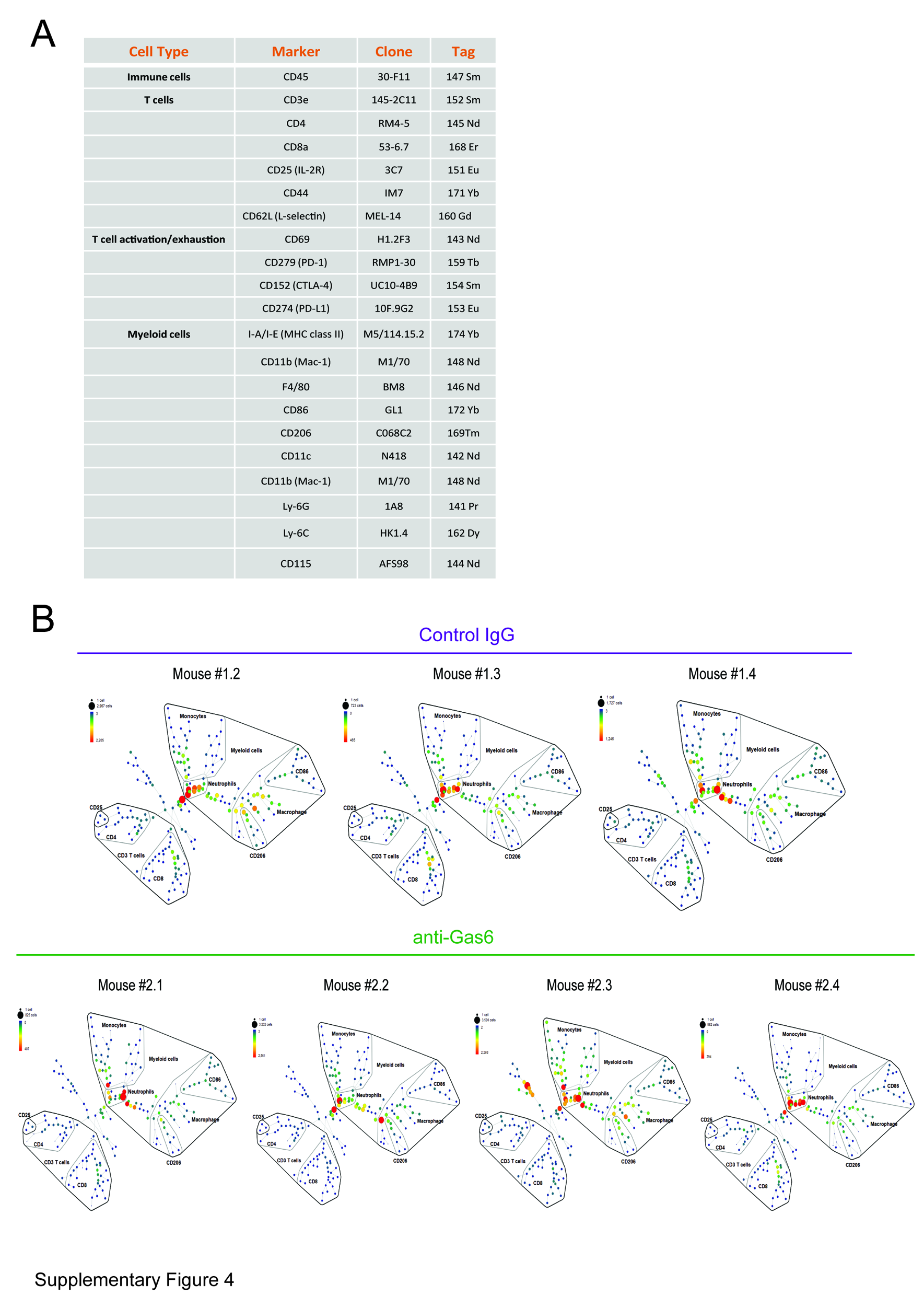

Supplement: Supplementary file 5 [file Image_4.TIF]

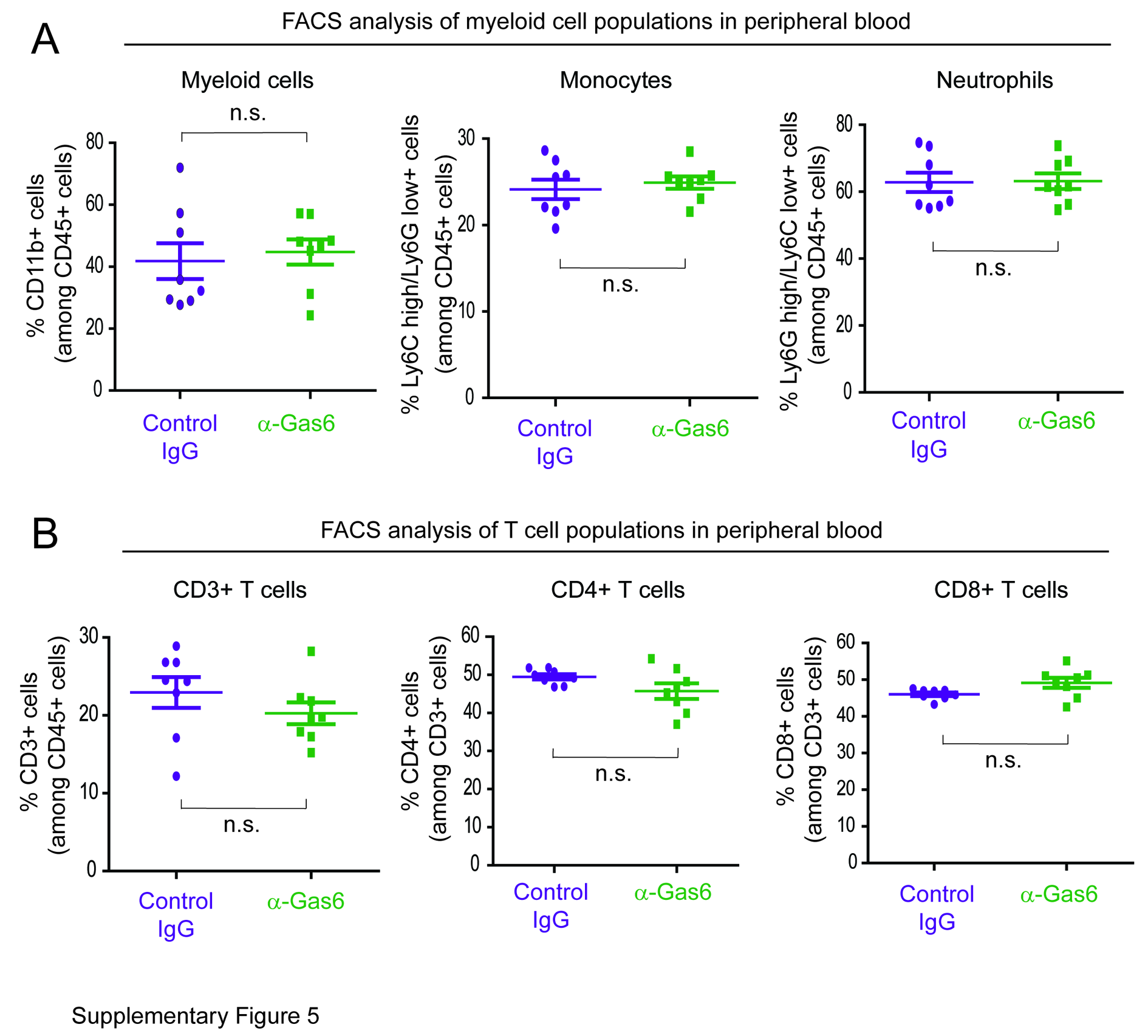

Supplement: Supplementary file 6 [file Image_5.TIF]

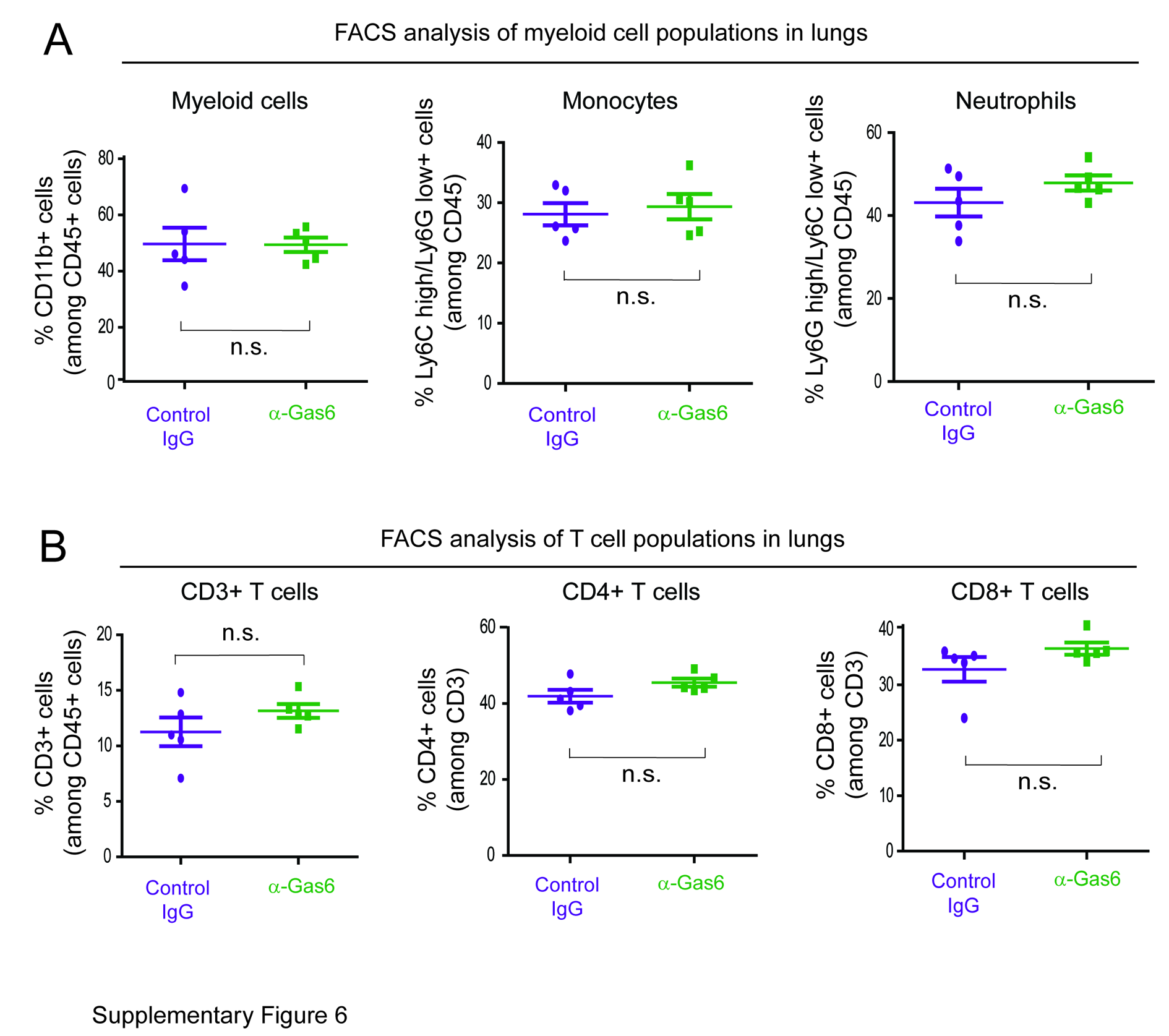

Supplement: Supplementary file 7 [file Image_6.TIF]

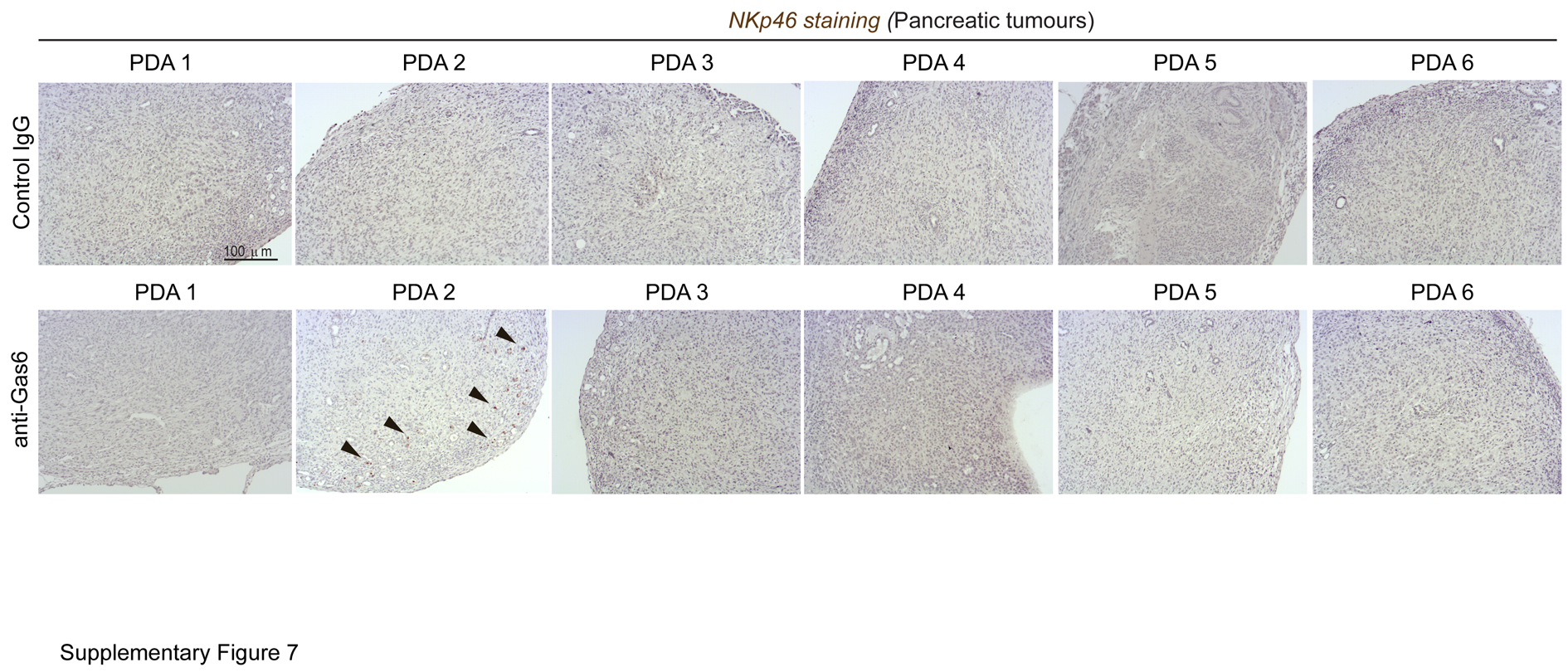

Supplement: Supplementary file 8 [file Image_7.TIF]

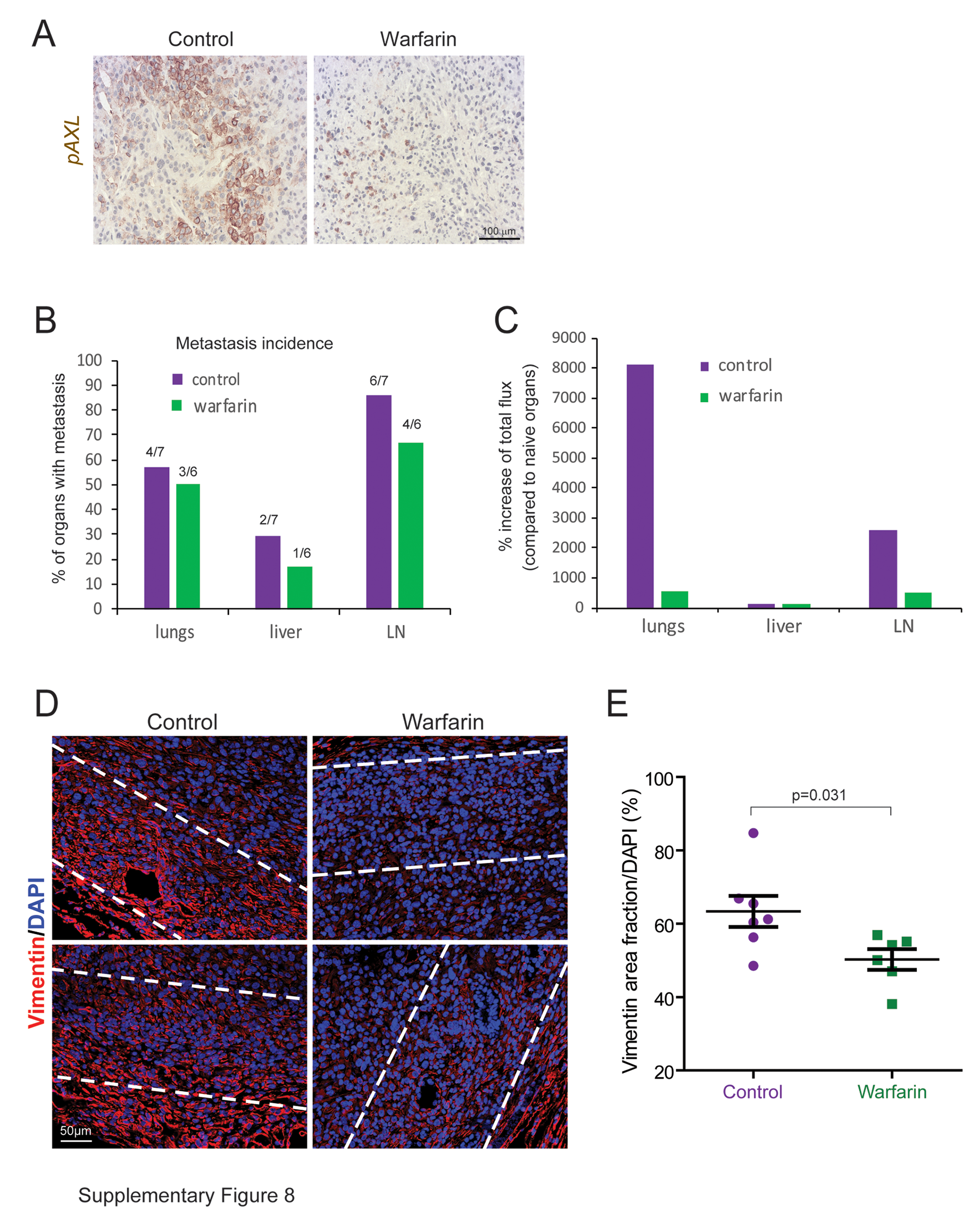

Supplement: Supplementary file 9 [file Image_8.TIF]
